# Traumatic Brain Injury in Mice Generates Early-Stage Alzheimer’s Disease Related Protein Pathology that Correlates with Neurobehavioral Deficits

**DOI:** 10.1007/s12035-024-04035-5

**Published:** 2024-02-27

**Authors:** Nicholas Panayi, Philip Schulz, Ping He, Brandon Hanna, Jonathan Lifshitz, Rachel K. Rowe, Michael R. Sierks

**Affiliations:** 1 Chemical Engineering, The School for Engineering of Matter, Transport and Energy, Arizona State University, Tempe, AZ 85287-6106 USA; 2 Department of Integrative Physiology, University of Colorado Boulder, Boulder, CO USA; 3 Department of Psychiatry, University of Arizona College of Medicine – Phoenix, Phoenix, AZ USA; 4 Phoenix Veteran Affairs Health Care System, Phoenix, AZ USA

**Keywords:** Beta-amyloid, Tau, Oligomers, Immunohistochemistry, Behavior, Concussion

## Abstract

Traumatic brain injury (TBI) increases the long-term risk of neurodegenerative diseases, including Alzheimer’s disease (AD). Here, we demonstrate that protein variant pathology generated in brain tissue of an experimental TBI mouse model is similar to protein variant pathology observed during early stages of AD, and that subacute accumulation of AD associated variants of amyloid beta (Aβ) and tau in the TBI mouse model correlated with behavioral deficits. Male C57BL/6 mice were subjected to midline fluid percussion injury or to sham injury, after which sensorimotor function (rotarod, neurological severity score), cognitive deficit (novel object recognition), and affective deficits (elevated plus maze, forced swim task) were assessed post-injury (DPI). Protein pathology at 7, 14, and 28 DPI was measured in multiple brain regions using an immunostain panel of reagents selectively targeting different neurodegenerative disease-related variants of Aβ, tau, TDP-43, and alpha-synuclein. Overall, TBI resulted in sensorimotor deficits and accumulation of AD-related protein variant pathology near the impact site, both of which returned to sham levels by 14 DPI. Individual mice, however, showed persistent behavioral deficits and/or accumulation of toxic protein variants at 28 DPI. Behavioral outcomes of each mouse were correlated with levels of seven different protein variants in ten brain regions at specific DPI. Out of 21 significant correlations between protein variant levels and behavioral deficits, 18 were with variants of Aβ or tau. Correlations at 28 DPI were all between a single Aβ or tau variant, both of which are strongly associated with human AD cases. These data provide a direct mechanistic link between protein pathology resulting from TBI and the hallmarks of AD.

**Supplementary Information:**

The online version contains supplementary material available at 10.1007/s12035-024-04035-5.

## Introduction

Traumatic brain injury (TBI) affects over two million people each year, and is now the principal cause of death among children and young adults [1, 2]. Although there is usually an initial resolution of physical symptoms following TBI, long-term cellular damage in the brain can occur, [3] leading to an increased susceptibility for a spectrum of neurodegenerative disorders [4]. Neurons are particularly vulnerable to the high sheer forces and mechanical deformation induced by TBI [5–9], where the resulting damage to protein transport mechanisms can result in axonal accumulation of neurofilament proteins, such as tau [9]. Aggregates of tau are also the major component of the hallmark neurofibrillary tangles in Alzheimer’s disease (AD) brain. In addition to changes in tau distribution, cellular stress induced by TBI can lead to a cascade of inflammatory processes in the brain and an increase in neuronal expression of both the amyloid precursor protein (APP) and beta-site APP cleaving enzyme (BACE-1) [10–12]. The increased expression of APP and BACE-1 increases amyloid-beta (Aβ) levels which can lead to toxic aggregation and accumulation of Aβ, also a characteristic feature of AD. Neuronal stress induced by TBI, in addition to changes in tau and Aβ distribution, can also lead to increased misprocessing, aggregation, and accumulation of alpha synuclein (α-syn) and TarDNA binding protein 43 (TDP-43) [1, 13]. Accumulation of misfolded or misprocessed variants of these four key neuronal proteins (tau, Aβ, α-syn, and TDP-43) is a common pathological feature underlying many neurodegenerative diseases in addition to AD (e.g., Amyotrophic lateral sclerosis (ALS), Parkinson’s disease (PD), Frontotemporal Lobular Degeneration (FTLD), and Dementia with Lewy Bodies (DLB)). However, the individual protein variants and brain locations of the hallmark protein deposits vary among these diseases [14–26]. Importantly, TBI leads to an increased incidence of this spectrum of neurodegenerative disorders [4].

The location, severity, and number of brain injuries can affect the evolution of different neurodegenerative diseases since the different pathogeneses involved may be attributed to the type and local environment of the neuronal cells damaged and ultimately the unique composition of toxic protein variants generated [1, 13, 27]. While the presence of different fibrillar protein aggregates such as amyloid plaques (Aβ), neurofibrillary tangles (tau), and Lewy bodies (a-syn) are characteristic and defining pathological features of individual neurodegenerative diseases such as AD, PD, FTLD and DLB, they may not represent the most effective early stage pathological markers for neurodegenerative disease. Rather, generation of small soluble toxic oligomeric variants of the different proteins likely precedes the formation of the hallmark fibrillar aggregates, and the presence of different combinations of these small soluble protein variants represent promising biomarkers for early detection of different neurodegenerative diseases. We previously developed a panel of single chain antibody variable domain (scFv) based reagents that very selectively bind toxic disease-related variants of tau, Aβ, TDP-43, and α-syn that are present in post-mortem validated human neurodegenerative disease brain tissue, but not in cognitively normal, age-matched human samples [28–33]. We have shown that this panel of reagents can be used to identify protein variant fingerprints that correlate with different neurodegenerative diseases, including AD, PD, FTD, and ALS [32, 34–38], and that a panel of reagents against AD associated protein variants can detect blood based biomarkers of AD during early, presymptomatic stages of AD [39]. We have also shown that these same protein variants are detected in the chronic TBI human brain, where respective protein variant fingerprints of blood samples taken from individual TBI cases mirror the protein variant fingerprint signature observed for specific neurodegenerative diseases [40].

We hypothesized that experimental TBI would generate distinct protein fingerprints of toxic neurodegenerative disease associated variants of tau, Aβ, TDP-43, and a-syn in individual mice, and that specific protein variants would correlate with injury-induced behavioral deficits. Here, we used a panel of neurodegenerative disease-selective reagents to histologically characterize brain tissue of mice subjected to diffuse TBI. We determined the extent of staining of each protein variant both to specific brain regions and as a function of time post-injury, and correlated individual anatomical protein variant staining levels with neurobehavioral performance. We show how both the spatial and temporal profiles of the neurodegenerative disease related variants that accumulate in the brain may help determine how the TBI associated neuronal stress contributes to AD and other long-term neurodegenerative disorders.

## Materials and Methods

### Rigor

All animal studies were conducted in accordance with the guidelines established by the internal IACUC (Institutional Animal Care and Use Committee) and the NIH guidelines for the care and use of laboratory animals. Studies are reported following the Animal Research: Reporting *In Vivo* Experiments (ARRIVE) guidelines. Randomization of animals was achieved by assigning individuals to treatment groups prior to initiation of the study to ensure equal distribution among groups. Data collection stopped at pre-determined final endpoints based on days post-injury (DPI). Animals were excluded from the study if post-operative weight decreased by ≥ 15% of pre-surgical weight or baseline rotarod score was not met. No animals were excluded based on these criteria. All animal behavior and histology were scored by investigators blinded to the treatment groups. Tissue samples analyzed in this study were generated from animals used in a previous study that non-invasively examined acute sleep after TBI [41].

### Animals

Male C57BL/6 mice (Harlan Laboratories, Inc., Indianapolis, IN) were used for all experiments. Mice were housed in a 14 h light:10 h dark cycle at a constant temperature (23 °C ± 2° C) with food and water available *ad libitum*. After surgery, mice were evaluated daily during post-operative care via physical examination and documentation of each animal’s condition. Animal care was approved by the Institutional Animal Care and Use Committee at the University of Arizona (13–460).

### Midline fluid percussion injury (mFPI)

Adult male mice (20-24 g; *n* = 52) were subjected to midline fluid percussion injury (mFPI) consistent with methods previously described [42–46]. Mice were anesthetized using 5% isoflurane in 100% oxygen for five minutes and the head of the mouse was placed in a stereotaxic frame with continuously delivered isoflurane at 2.5% via nosecone. While anesthetized, body temperature was maintained using a Deltaphase® isothermal heating pad (Braintree Scientific Inc., Braintree, MA). A midline incision was made exposing bregma and lambda, and fascia was removed from the surface of the skull. A trephine (3 mm outer diameter) was used for the craniectomy, centered on the sagittal suture between bregma and lambda without disruption of the dura. An injury cap prepared from the female portion of a Luer-Loc needle hub was fixed over the craniotomy using cyanoacrylate gel and methyl-methacrylate (Hygenic Corp., Akron, OH). The incision was sutured at the anterior and posterior edges and topical Lidocaine ointment was applied. The injury hub was closed using a Luer-Loc cap and mice were placed in a heated recovery cage and monitored until they were ambulatory before being returned to their home cages.

For injury induction 24 h post-surgery, mice were re-anesthetized with 5% isoflurane delivered for three minutes. The cap was removed from the injury-hub assembly and the dura was visually inspected through the hub to ensure it was intact with no debris. The hub was then filled with normal saline and attached to an extension tube connected to the male end of the fluid percussion device (Custom Design and Fabrication, Virginia Commonwealth University, Richmond, VA). An injury of moderate severity (1.4 atm) was administered by releasing the pendulum onto the fluid-filled cylinder following the return of a toe-pinch response. Sham-injured mice underwent the same procedure except the pendulum was not released. The injury hub was removed, and the brain was inspected for uniform herniation and integrity of the dura. The incision was cleaned with saline and closed using sutures. Mice received either sham injury, a single TBI, or two TBIs (3–9 h injury interval). For mice that received two TBIs, the injury hub was left intact following the first TBI and the mouse was reattached to the injury device for a second TBI before the hub was removed, the dura was inspected, and the incision was closed. To control for the anesthetic used during the induction of injury, uninjured shams and the single TBI group also received a second exposure to isoflurane. After spontaneously righting, mice were placed in a heated recovery cage and monitored until they were ambulatory before being returned to their cage. Adequate measures were taken to minimize pain or discomfort.

### Rotarod

Sensorimotor function was assessed using a rotarod (Economex Rotarod, Columbus Instruments, Columbus, OH). Mice were acclimated to the apparatus and trained for three days prior to the craniectomy surgery. During the acclimation phase, mice were placed on the stationary rod and allowed to explore for 30 s. The mice were trained by placing them on the rod at a constant speed of five revolutions per minute (rpm) until the mice could walk 15 s at five rpm. Next, mice were placed on the rod with an initial rotation speed of five rpm and an acceleration of 0.2 rpm/sec. The trial ended when the mouse fell off the rod; the acclimation period ended after two trials. Mice were trained over three consecutive days and the last training session was recorded as a baseline score. Testing occurred at 2, 5, and 7 DPI. For the training and testing phase, two trials were run back-to-back and mice were returned to cages thereafter. After 10 min, mice had a third trial. Time spent on the rotarod from the best two trials were averaged to generate a time score for each mouse.

### Neurological Severity Score (NSS)

Sensorimotor function and balance were assessed using a modified neurological severity score at 2, 5, and 7 DPI. Mice were observed for hind limb flexion, startle reflex, and seeking behavior (presence of these behaviors was considered successful task completion). Mice traversed in sequence, three, two, and one-centimeter beams. The beams were elevated, and mice were given one minute to travel 30 cm. The task was scored as a success if the mouse traveled 30 cm without a forelimb/hindlimb hanging from the beam. Mice were also required to balance on a 0.5-cm beam and a 0.5-cm round rod for three seconds in a stationary perched position (front paws between hind paws). One point was given for failure on an individual task, whereas no points were given if a mouse completed a task successfully. A composite score was assigned to each mouse that ranged from zero to eight. A high composite NSS score is indicative of task failure and interpreted as neurological impairment.

### Elevated Plus Maze (EPM)

Anxiety-like behavior was assessed using the elevated plus maze. Mice were placed at the junction of the four arms, facing an open arm, and given five minutes to explore the apparatus. The elevated plus maze consisted of two open arms and two arms with high walls. Mice were visually recorded using an overhead camera and recordings were analyzed using EthoVision XT 10 software. Time spent in the open arms and the closed arms were calculated. Open arm activity was interpreted as anti-anxiety behavior.

### Forced swim task (FST)

Depressive-like behavior was assessed using the FST. Mice were placed into glass cylinders (15 cm diameter × 24.5 cm high) filled with water (25º C) for six minutes. The first minute was excluded from analysis as an acclimation phase. Mice were visually recorded using an overhead camera and recordings were analyzed using EthoVision XT 10 software. Time spent actively swimming and time spent immobile was calculated for each mouse.

### Novel Object Recognition (NOR)

Cognitive impairment was tested using the NOR test as previously published [45, 47]. The test consisted of three phases: habituation, training, and testing. On the day of the test, mice were placed in an open field (42 × 21 × 21 cm) for one hour of habituation. Mice were removed from the open field and two identical objects were placed in opposing corners for the training phase. Mice were placed in the center of the field and given five minutes to explore the objects. Following training, mice were returned to their home cages for four hours. For the testing phase, one familiar object was placed in the original location and one novel object was placed in the opposing corner. Mice were placed into the center and given five minutes to explore. For testing, the times spent actively investigating the novel and familiar object were quantified. Investigation of an object included the mice sniffing, touching, or climbing onto an object while facing the object. A discrimination index (DI) was calculated for each mouse: ${\mathrm{DI}}=\frac{{\mathrm{T}}{\mathrm{novel}} \, }{{\mathrm{T}}{\mathrm{novel}}\mathrm{ + T}{\mathrm{familiar}}}\times 100$.

### Tissue preparation

Following behavioral assessment, brain tissue was collected and brain pathology was characterized as a function of TBI and time post-injury. Brain tissue was harvested at 7, 14, or 28 DPI. Mice were given an overdose of sodium pentobarbital intraperitoneally and transcardially perfused with a phosphate buffered saline (PBS) flush followed by 4% paraformaldehyde. The brains were transferred to fresh fixative solution and sent to NeuroScience Associates Inc. (Knoxville, TN) to be processed and sectioned. Brains were embedded into a gelatin matrix where they could be frozen and sectioned from one solid block (MultiBrain® Technology, NeuroScience Associates). Sections of 40 μm thickness were taken in the coronal plane and returned to the laboratory for free floating staining.

### ScFv-Phage production

The seven different scFvs utilized (Table 1) [28–31, 33] were each expressed on the surface on bacteriophage as described. Briefly, the expression plasmids encode the scFv connected to the M13 phage minor coat protein pIII, for expression of the scFv on the phage surface. TG1 cells containing the plasmids were incubated with M13 hyperphage (Progen, Germany) or KM13 helper phage overnight to generate the scFv-phage fusion construct. Scfv fusion phage were purified by repeated polyethylene glycol (PEG 8000) precipitation and centrifugation.

**Table 1:** List of scFvs and their target antigens

scFv	Target Antigen Description	Validated targets
Tau variants		
F9T	Synthetically generated trimeric tau variant	Human AD brain tissue, blood samples, neuronal cell culture, AD mouse model, human TBI blood samples [39, 40, 61]
D11C	Synthetically generated trimeric tau variant	Human AD brain tissue, blood samples, neuronal cell culture, AD mouse model, human TBI blood samples [37, 39, 40, 61]
Aβ variants		
A4	Synthetically generated oligomeric Aβ	Human AD brain tissue, blood samples, human TBI blood samples, AD mouse model [33, 37–40, 48]
C6T	Human AD brain derived oligomeric Aβ	Human AD brain tissue, blood samples, human TBI blood samples, AD mouse model [37–40, 48, 59]
α-synuclein variants		
10H	Small in vitro generated oligomers	Human PD brain tissue, blood samples, human TBI blood samples [28, 29, 38, 63]
D5	Small in vitro generated oligomers	Human PD brain tissue, blood samples, human TBI blood samples[28, 29, 38, 63]
TDP-43 variants		
AD-TDP3	Human AD and ALS brain derived TDP-43 variant	Human AD brain tissue, blood samples, human TBI blood samples [34, 39, 40]

### Chromogenic DAB Immunostaining

**Fig. 1:**
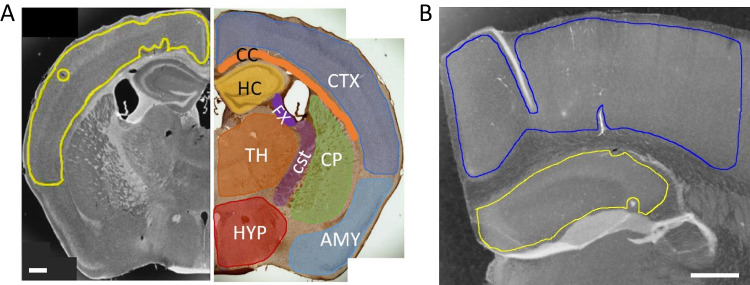
Each brain image was divided into 7–10 generalized regions of interest (ROI), depending on which regions were present in each brain slice. Shown: Upper Cortex CTX (blue); corpus callosum CC (orange); hippocampus HC (yellow); caudoputamen CP (green); fornix FX (purple); corticospinal tract CST (magenta); thalamus TH (orange); hypothalamus HYP (red); amygdala/olfactory/cortical subplate AMY (light blue). Striatum STR is located rostrally and is not clearly visible in this slice. Non-measurable areas including edges, wrinkles, overlaps, tears, and/or contaminants were excluded from the ROI selections. Scale bar 500 µm

Brain tissue was stained for the presence of different variants of Aβ, tau, TDP-43, and α-syn utilizing a panel of seven different scFvs [28–31, 33], where each scFv binds a different neurodegenerative disease related protein variant (Table 1), and a commercial antibody against Aβ (6E10, Biolegend). Brain sections of each mouse were separately probed with each of the seven scFvs or commercial Aβ antibody, and staining intensities of 10 different brain regions were determined (Fig. 1A). Brain sections were also probed for IgG using anti-mouse IgG horse antibody (Vector Labs).

Immunostaining was performed as described previously [48, 49]. Tissue sections underwent a 10-min-high-temperature antigen retrieval step and incubated with scFv-phage (1:1000) for one hour; then placed in anti-M13 mouse antibody (Invitrogen 1:4000) incubation for one hour. Sections were washed and incubated with biotinylated anti-mouse IgG horse antibody (1:1000). Following washing, the Vectastain ABC-HRP kit (Vector Laboratories, Burlingame, CA) was applied. Samples were visualized using 3,3′-diaminobenzidine (DAB) as a substrate (Vector Laboratories). Sections were counterstained with hematoxylin. Since all 39 mice brain slices were embedded into a single MultiBrain slice, staining intensities of each brain slice could be compared.

### Immunostaining Image Analysis

Brightfield images were collected at 4x, 10x, and 40x using a Keyence BZ-X800 and a Leica TCS SP5 LSCM or EVOS M7000 in the Regenerative Medicine Imaging Facility at ASU. 4x image stitching was performed using Keyence software or NIH Image J 1.52p (FIJI 2). Antibody staining was measured using NIH ImageJ with a measured mean grey value intensity (integrated densitometry/area) of phage staining ranging from 0–255, (0 = no stain; 255 = hypothetical high) for selected regions of interest. We categorized each coronal section into different regions of interest including: cortex (CTX); corpus callosum (CC); hippocampus (HC); caudoputamen (CP); fornix (FX); corticospinal tract (CST); striatum (STR); thalamus (TH); amygdala/olfactory/cortical subplate (AMY). Not all sections contained all regions, such that 6–8 regions were defined for each section. Due to the high temperature antigen retrieval step and the size of the multi-brain slices, adherence to the slides caused some separations and wrinkles in the tissue. Care was taken when analyzing tissue to avoid anomalous regions where tissue had tears or wrinkles, or areas where tissue overlapped (Fig. 1B). Analyses with the C6T (Aβ) scFv also included the percentage of region covered by clustered punctate staining. Hematoxylin nuclear staining was removed in Image J using the LAB Color Threshold option, then a black and white threshold was used to eliminate the diffuse gradient staining. The punctate C6T (Aβ) DAB staining was selected and the percentage of stained area (ImageJ: Area Fraction) within the selected regions of interest was calculated.

### Statistical Analyses

Statistical analyses were performed in SPSS 25 (Chicago, IL, USA) and Graphpad Prism 9.5.1 (San Diego, California USA) software. Values for each antibody staining image were analyzed either as raw values or normalized to the average sham values. Uninjured shams from each time point were pooled into a single control group. In order to determine the significant intergroup difference, one-way analysis of variance (ANOVA) was used followed by a multiple comparison analysis using a Dunnett post hoc test. Statistical significance was classified as *p* < 0.05.

Correlations between behavioral scores obtained at a specific DPI and scFv staining results obtained from brain tissue harvested at the same DPI were performed. Behavioral scores obtained at 2, 5, and 7 DPI were correlated with scFv staining levels from brain tissue harvested at 7 DPI. The ***r***^***2***^ values were generated by two tail Pearson bivariate correlation in SPSS.

## Results

### Diffuse TBI resulted in acute sensorimotor deficits and delayed onset of depressive-like behavior

**Fig. 2:**
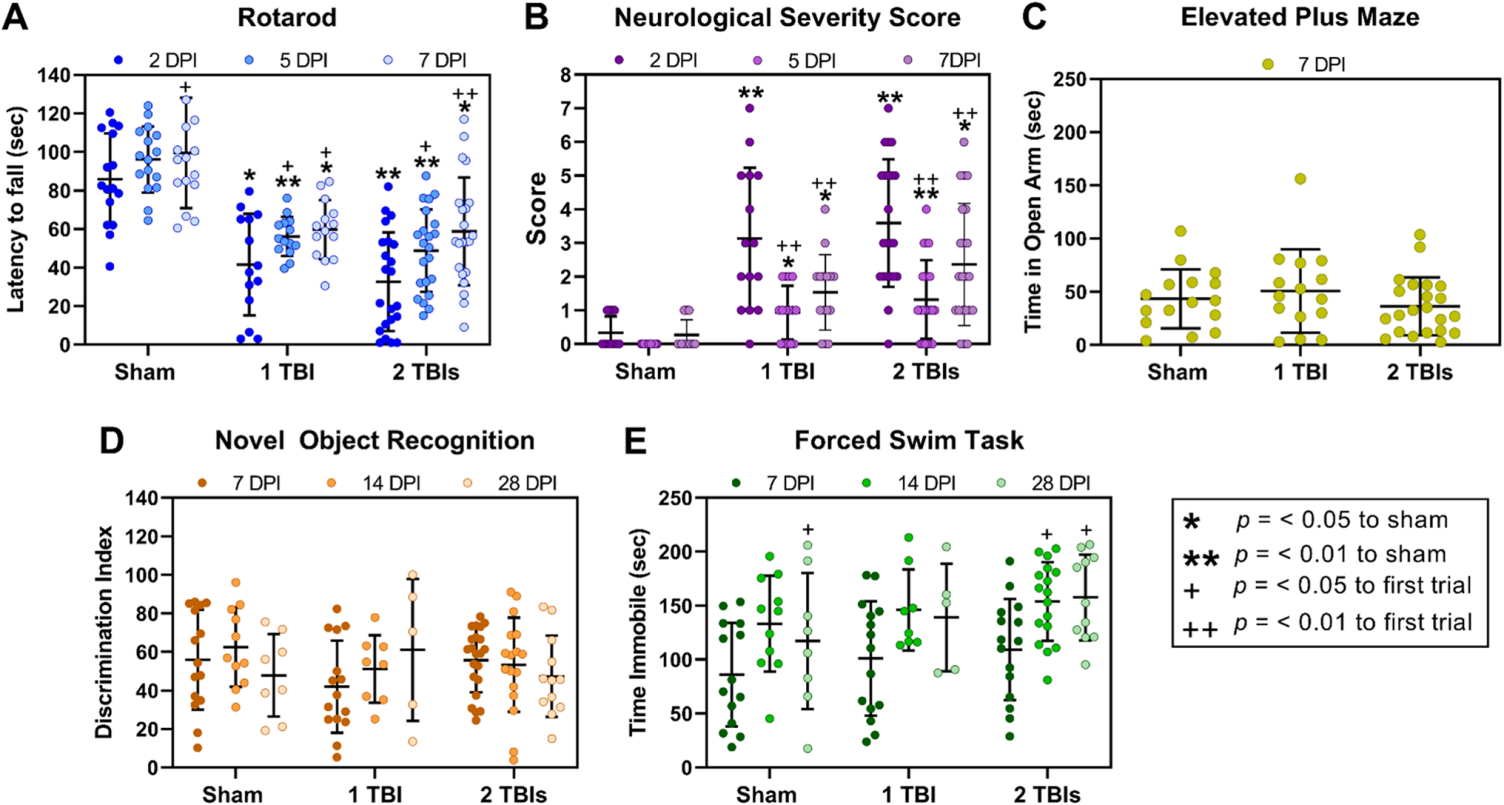
Diffuse TBI resulted in behavioral deficits. **A** Latency to fall from the rotarod measured at 2, 5, and 7 days post-injury (DPI). Mice subjected to 1 TBI or 2 TBIs had significantly shorter latencies to fall at all time points. **B** Sensorimotor deficits were assessed with a neurological severity score (NSS). Mice subjected to 1 TBI or 2 TBIs had significantly higher scores at all time points compared to uninjured shams. **C-D** There were no brain injury-induced deficits on the elevated plus maze or the novel object recognition task. **E** TBI did not result in anxiety-like behavior assessed with the forced swim task (FST). There were no injury-induced changes to the time spent immobile during the FST. However, there were significant time effects and mice spent more time immobile in subsequent trials compared to the first trial. Data are presented as individual data points with the mean and SEM

Mice were subjected to either one or two TBIs to model single (1 TBI) or repetitive (2 TBIs) brain injury, or to a control sham injury (sham). Diffuse TBI resulted in a significant injury and time effect on rotarod performance (Fig. 2A). Mice in the 1 TBI and 2 TBIs groups had a shorter latency on the rotarod at 2, 5, and 7 DPI compared to uninjured shams (F_2, 49_ = 31.01, *p* < 0.0001). All mice showed improvement on the rotarod as a function of time post-injury (F_2, 98_ = 19.59, *p* < 0.0001). Mice in the 1 TBI and 2 TBIs groups had higher scores on the neurological severity score (NSS) task compared to shams (Fig. 2B). Brain-injured mice had a higher NSS score at 2, 5, and 7 DPI compared to uninjured shams (F_2, 49_ = 16.86, *p* < 0.0001). Brain-injured mice showed improvement as a function of time post-injury (F_2, 98_ = 45.84, *p* < 0.0001) with lower scores on 5 and 7 DPI compared to 2 DPI. There was no significant injury effect (F_2, 50_ = 0.58, *p* = 0.56) on the time spent in the open arms of an elevated plus maze (Fig. 2C). There was also no injury effect (F_2,104_ = 0.28, *p* = 0.76) nor time effect (F_2, 104_ = 0.40, *p* = 0.67) on the discrimination index from the novel object recognition (NOR) task (Fig. 2D). There was an overall significant injury effect on time spent immobile in the forced swim task (F_2, 95_ = 3.26, *p* = 0.04; Fig. 2E). There was also a significant effect of time after injury (F_2, 95_ = 10.55, *p* < 0.0001) where sham mice spent more time immobile at 28 DPI compared to 7 DPI, and mice in the 2 TBIs group spent more time immobile at 14 DPI and 28 DPI compared to 7 DPI.

### Diffuse TBI resulted in the accumulation of toxic protein variants in brain tissue

**Fig. 3:**
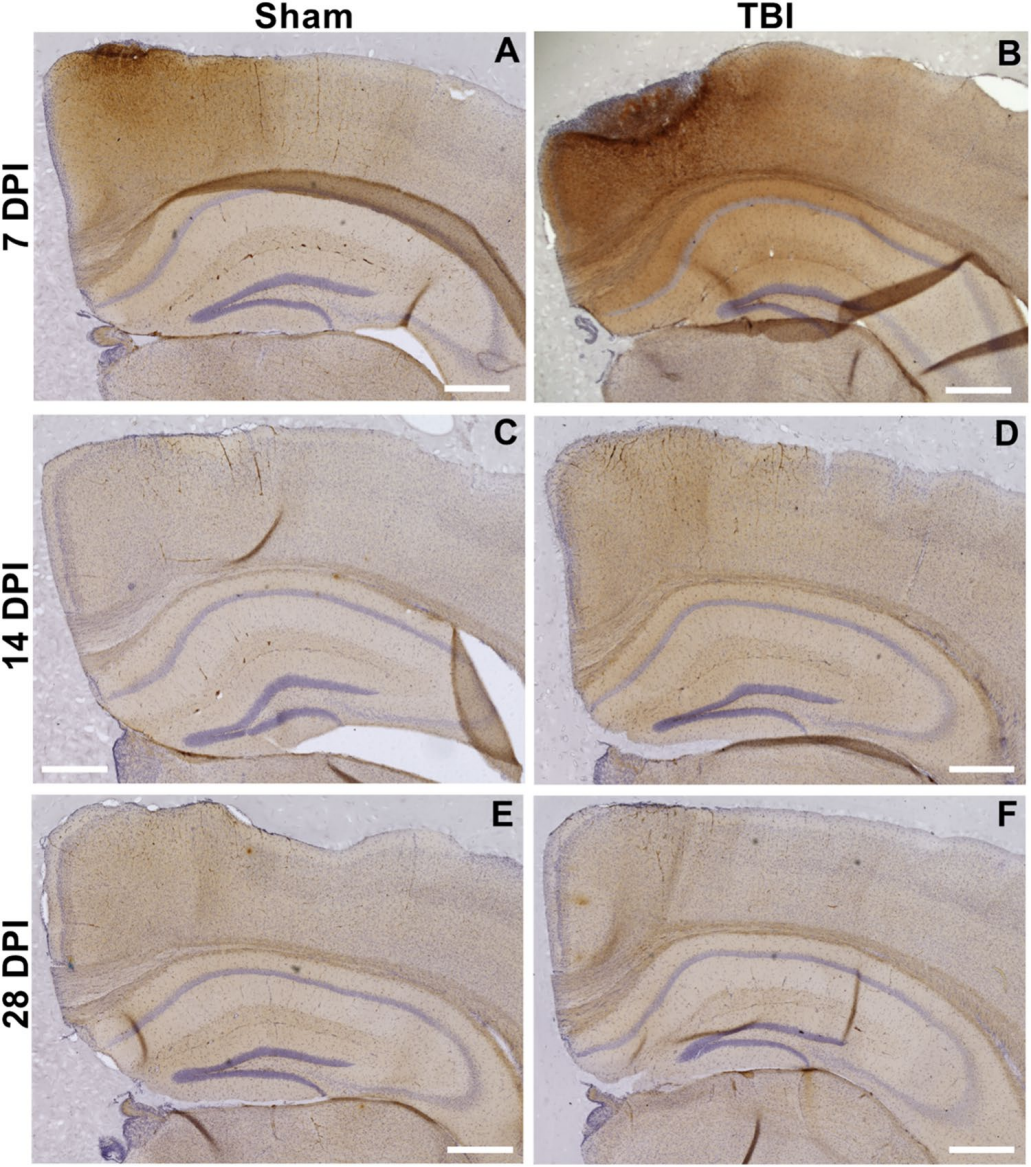
Representative coronal mice brain staining detected with D11C scFv-phage grouped by Sham (craniectomy without TBI; left) or TBI (right). Tissue was collected at **(A-B)** 7, **(C-D)** 14, or **(E–F)** or 28 days post-injury (DPI). Phage were detected with HRP conjugated secondary/DAB (brown) and nuclei detected with hematoxylin (purple). Phage staining was detected throughout the brain, though most intense staining was observed near the site of craniectomy and TBI in tissue collected at 7 DPI. The presence of the target protein variant resolved by 14 and 28 DPI. Scale bar 500 µm

Representative images of D11C (tau) scFv staining of coronal brain sections from sham mice (craniectomy only) and mice subjected to TBI are shown at 7, 14, or 28 DPI (Fig. 3). Dense staining of all protein variants was observed around the site of the craniectomy and brain injury at 7 DPI in both sham and TBI mice (Fig. 3A, B) reflecting a combination of IgG staining from localized blood brain barrier damage and high local generation and accumulation of the protein variants. Staining for monomeric Aβ with the commercial antibody 6E10 showed a similar result (Supplemental Fig. 1). Similarly, there was significant accumulation of IgG stain in tissue and blood vessels around the site of injury 7 DPI (Supplemental Fig. 2). Representative high resolution images show localized F9T (tau) and C6T (Aβ) staining of coronal brain sections at 7 DPI (Supplemental Fig. 3A and 3B). The localized staining near the brain injury impact site was transient and resolved by 14 and 28 DPI for all the scFvs and for IgG and monomeric Aβ (Fig. 3 C-F, and Supplemental Figs. 1B, 2B).

**Fig. 4:**
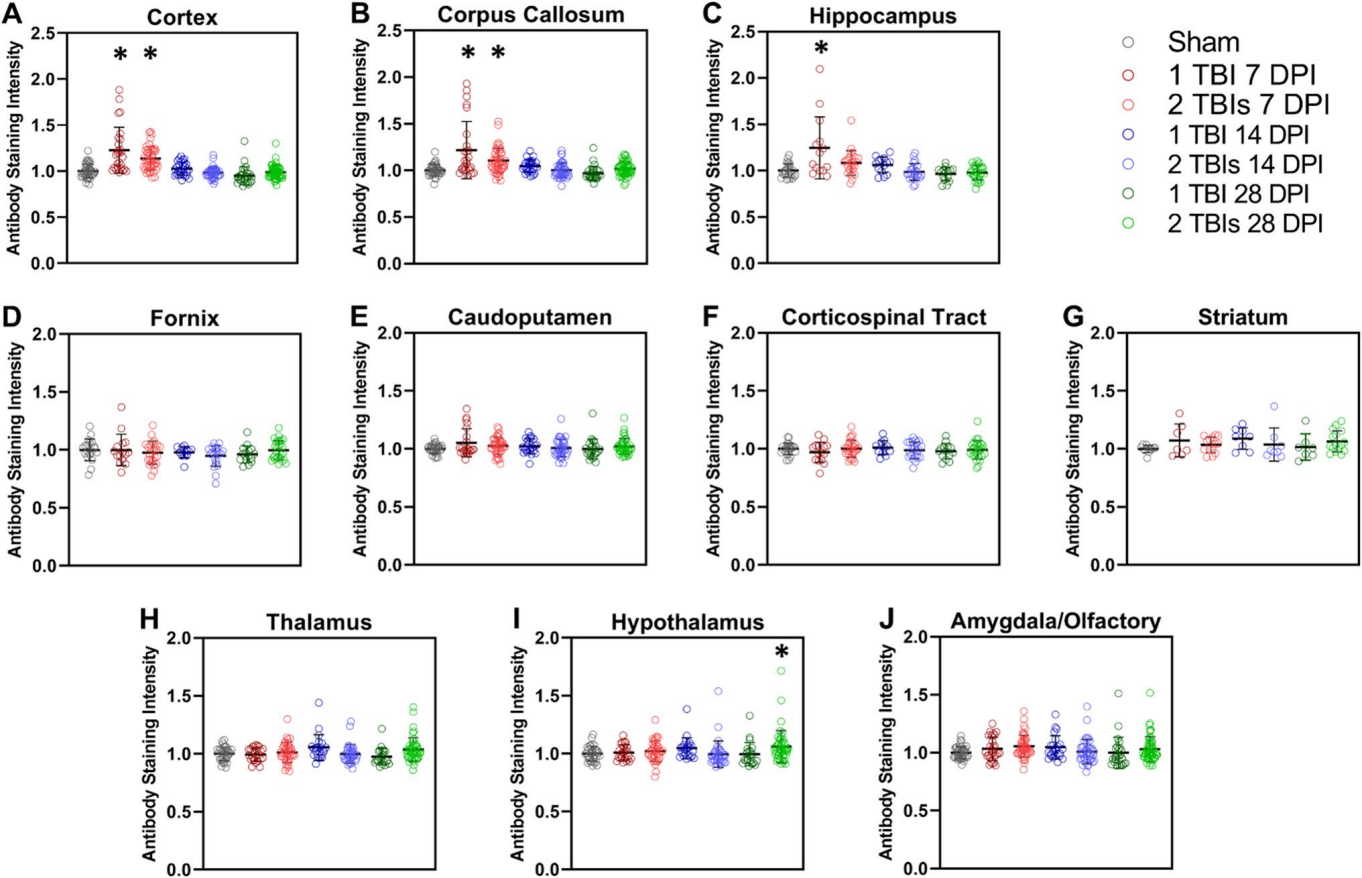
Combined staining intensity values of each of seven scFv phage in 10 different brain tissue regions. Brain tissue images from each individual mouse were converted to 8-bit black and white images and inverted so phage staining correlated to brightness (0–255). Mean grey value intensity (brightness) of each brain region of interest was measured by ImageJ analysis. Values were normalized to the average grey value intensity of the corresponding brain region in sham treated mice to generate the combined values for each of the seven scFv phage. Brain tissue was collected at 7, 14, or 28 days post-injury (DPI). Mice were subjected to either a control sham injury, 1 TBI, or 2 TBIs. Post hoc analysis of composite staining intensity of all seven scFvs showed statistically significant differences in the composite staining intensity at 7 DPI in TBI versus sham mice in the cortex (1 TBI *p* < 0.0001, 2 TBIs *p* < 0.0001), the corpus callosum (1 TBI *p* < 0.0001, 2 TBIs *p* = 0.002) and hippocampus (1TBI *p* < 0.0001). These differences resolved by 14 and 28 DPI. The only composite protein variant staining intensity that did not resolve by 14 DPI was in hypothalamus of mice subjected to 2 TBIs (*p* = 0.037). We also observed unresolved elevated levels of protein variants in other deeper brain regions among individual outlier cases, but these did not reach statistical significance. Asterisks indicate *p* < 0.05 significance compared to corresponding sham region. Error bars are ± SD

A composite plot of all seven scFvs (Aβ, tau, a-syn, and TDP-43) in each of the 10 different brain regions, for each treatment cohort, indicates the overall scFv staining intensities (Fig. 4). Results reflected the same time dependent distribution where the composite staining intensity at 7 DPI was substantially higher in regions with a closer proximity to the site of injury (Fig. 4A-C), and lower in brain regions more distant from the site of injury (Fig. 4D-J). One-way ANOVA analyzing the composite staining intensity showed significant differences in the cortex (CTX; F_6,261_ = 25.26, *p* < 0.0001; Fig. 4A), corpus callosum (CC; F_6,252_ = 13.21, *p* < 0.0001; Fig. 4B), hippocampus (HC; F_6,136_ = 8.46, *p* < 0.0001; Fig. 4C), thalamus (TH; F_6,220_ = 2.81, *p* = 0.012; Fig. 4H) and hypothalamus (HYP; F_6,220_ = 2.41, *p* = 0.029; Fig. 4I). There were no differences in staining intensity in the fornix (FX; F_6,130_ = 0.98, *p* = 0.44; Fig. 4D), caudoputamen (CP; F_6,210_ = 1.52, *p* = 0.174; Fig. 4E), corticospinal tract (CST; F_6,132_ = 0.59, *p* = 0.74; Fig. 4F), striatum (ST; F_6,64_ = 1.01, *p* = 0.43; Fig. 4G), or amygdala/olfactory/cortical subplate (AMY; F_6,251_ = 1.73, p = 0.12; Fig. 4J). Dunnett post hoc analysis indicated significant differences in the 1 TBI group and the 2 TBIs group at 7 DPI in both the CTX (Fig. 4A) and CC (Fig. 4B) compared to sham. The 1 TBI group had higher staining intensity at 7 DPI in the HC compared to sham (Fig. 4C). The 2 TBIs group had higher staining intensity in the HYP at 28 DPI compared to the sham group (Fig. 4I).

**Fig. 5:**
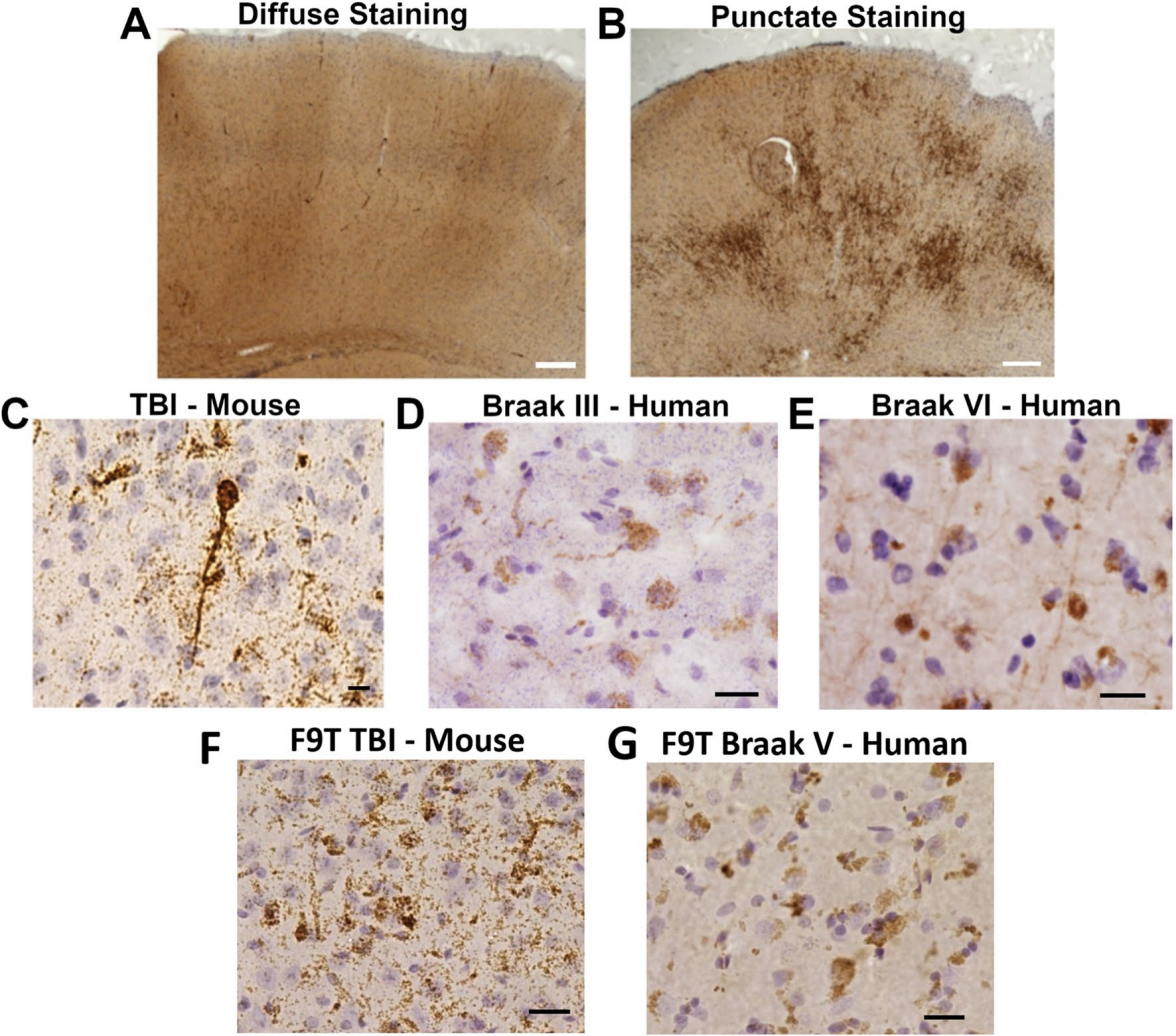
Staining with the C6T-phage displayed two distinct patterns, (**A**) a diffuse staining pattern observed with the other reagents, and (**B**) a punctate staining pattern unique to C6T phage. Brain tissue was incubated with C6T- or F9T-phage, detected with peroxidase conjugated secondary and stained with DAB. Staining of mouse cortex tissue shown here. At higher magnification, the C6T Aβ variant punctate staining pattern is observed in: (**C**) cytoplasmic staining of neurons in individual TBI mice, and in human post-mortem (**D**) Braak stage III, and (**E**) Braak stage VI AD brain tissue. F9T tau variant staining pattern is observed in: (**F**) cytoplasmic staining of neurons in individual TBI mice, and in human post-mortem (**G**) Braak stage V AD brain tissue. White Scale bar 200 µm, black scale bar 20 µm

The individual protein variant staining intensity levels in different brain regions, quantified as a function of time post-injury, showed similar trends to those observed in the composite staining intensities presented in Fig. 4 (for full results see Supplemental Table 1–8 and Supplemental Figs. 4-7). The C6T (Aβ) scFv, which binds an AD brain derived oligomeric Aβ variant, had two distinct staining patterns, a diffuse staining pattern similar to that observed with the other scFvs (Fig. 5A), and a punctate staining pattern that was observed to varying degrees in all the mice (Fig. 5B). These two staining patterns were analyzed separately (Supplemental Table 2, 3 and Supplemental Fig. 4B, C). Significant differences in the CTX, the region of the brain directly below the site of TBI, were observed for all protein variants except the TDP-43 protein variant (ADTDP3 scFv) between the 7 DPI and sham groups (Supplemental Figs. 4-7). Several other significant differences in the 7 DPI group were also observed with selected protein variants in other brain regions near the site of injury including the CC and HC (Supplemental Figs. 4A, 5B and 6A). Only one significant difference in protein variant staining in a brain region further from the site of injury was observed, with the D11C (tau) variant between the 28 DPI and sham groups (Supplemental Fig. 5B).

### Protein variant levels correlated to TBI-induced behavioral deficits

Both protein variant levels and behavioral data showed considerable variation from mouse to mouse in all the cohorts studied including the sham group which received a craniectomy but no TBI. To determine if protein variant and behavioral data variations are linked, we correlated levels of the seven different protein variants in each of the 10 brain regions of interest in individual mice from all the groups with behavior scores measured on the same DPI. We separately correlated the C6T (Aβ) diffuse and punctate staining levels. There were 21 statistically significant correlations (*p* < 0.05) between protein variants in specific brain regions and specific behavioral deficits: 13 at 7 DPI, one at 14 DPI, and seven at 28 DPI (Table 2). Of the 13 correlations with acute behavioral deficits measured at 7 DPI, eight were with the A4 (Aβ) variant, two with the D11C (tau) variant, two with the AD-TDP3 (TDP-43) variant, and one with the D5 (α-syn) variant (Table 2). All of the 13 correlations observed at 7 DPI resolved by 14 DPI, where the only significant correlation was with the A4 (Aβ) variant (Table 2).

**Table 2:** Protein variants detection by brain region correlated with age-matched behavior scores days post-injury (DPI) associated with cognitive-decline

DPI	r^2^	p	Behavior test and DPI	ScFv & Brain Region	Target Antigen	Number of mice (n)
7 DPI	0.785	0.0187	RR 2DPI	ADTDP3 CC	TDP-43	10
	0.783	0.0015	NOR 7DPI	D11C AMY	Tau	10
	0.734	0.0292	FST 7DPI	D5 CC	α-syn	3
	0.666	0.0477	RR 5DPI	ADTDP3 CC	TDP-43	3
	0.630	0.0061	NOR 7DPI	D11C TH	Tau	5
	0.624	0.0065	RR 5DPI	A4 TH	Aβ	10
	0.593	0.0092	RR 2DPI	A4 HYP	Aβ	9
	0.558	0.0130	RR 2DPI	A4 AMY	Aβ	10
	0.517	0.0191	RR 5DPI	A4 HYP	Aβ	5
	0.517	0.0191	RR 5DPI	A4 FF	Aβ	10
	0.501	0.0221	NSS 2DPI	A4 AMY	Aβ	7
	0.459	0.0313	RR 2DPI	A4 FF	Aβ	4
	0.428	0.0400	RR 2DPI	A4 TH	Aβ	9
14 DPI	0.405	0.0480	NOR 14DPI	A4 AMY	Aβ	10
28 DPI	0.436	0.0073	FST 28DPI	F9T CC	Tau	15
	0.429	0.0080	FST 28DPI	F9T HYP	Tau	15
	0.335	0.0486	FST 28DPI	F9T CST	Tau	12
	0.311	0.0384	NOR 28DPI	C6%area CC	Aβ	14
	0.307	0.0320	FST 28DPI	C6%area HYP	Aβ	15
	0.304	0.0330	FST 28DPI	C6T HYP	Aβ	15
	0.274	0.0452	NOR 28DPI	C6T CTX	Aβ	15

Correlations with significant Pearson correlation (p < 0.05) ranked by r^2^ value.

**Fig. 6:**
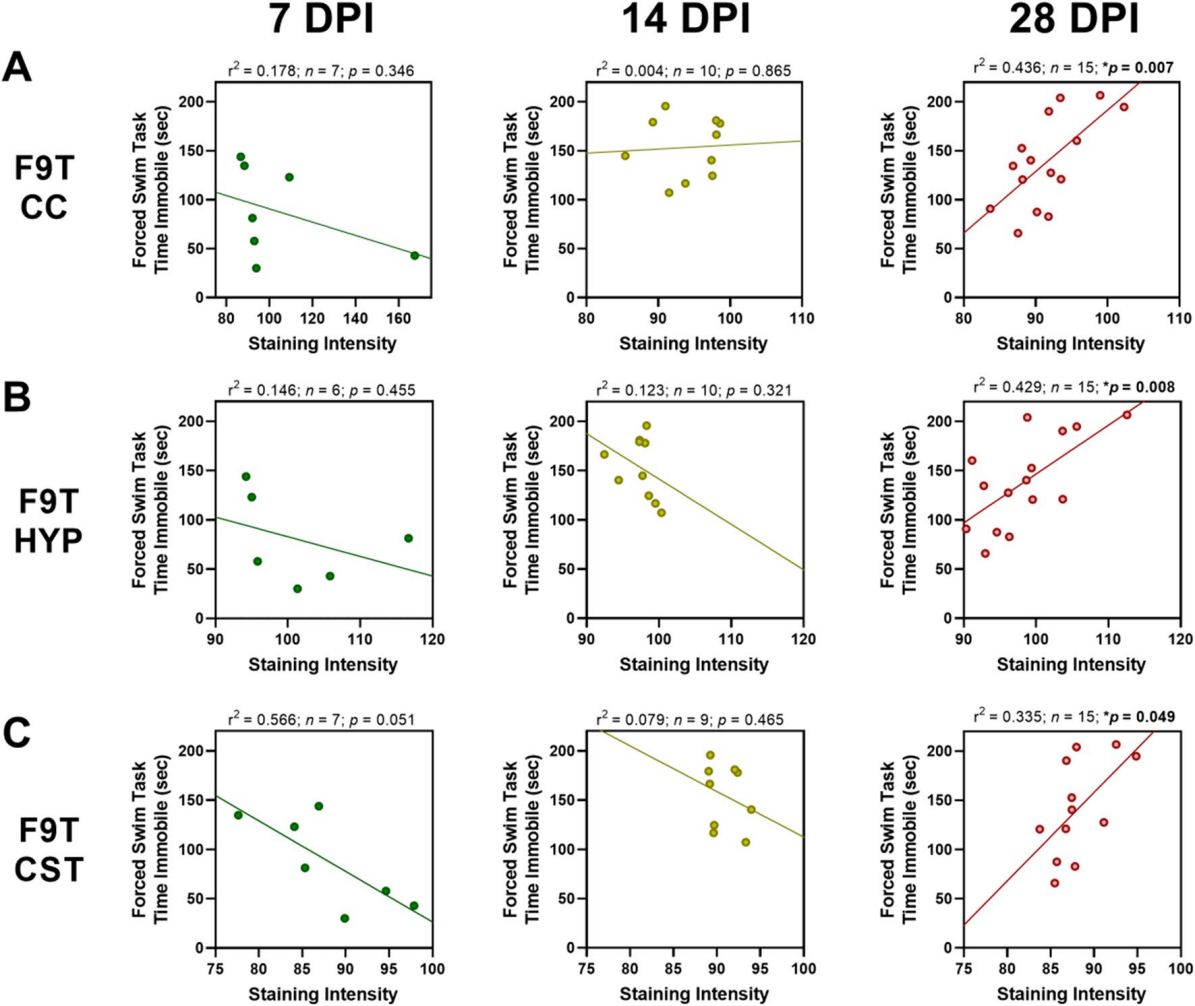
Negative correlations between time spent immobile during the forced swim task (FST) and F9T tau variant staining level increased with days post-injury (DPI). At 7 and 14 DPI, there were no statistically significant correlations with staining in the (**A**) corpus callosum (CC), (**B**) hypothalamus (HYP), or (**C**) corticospinal tract (CST). At 28 DPI, a significant correlation with behavioral deficits in the FST was observed. Levels of F9T tau variant showed a positive correlation with FST at 7 DPI, but a strong negative correlation by 28 DPI

**Fig. 7:**
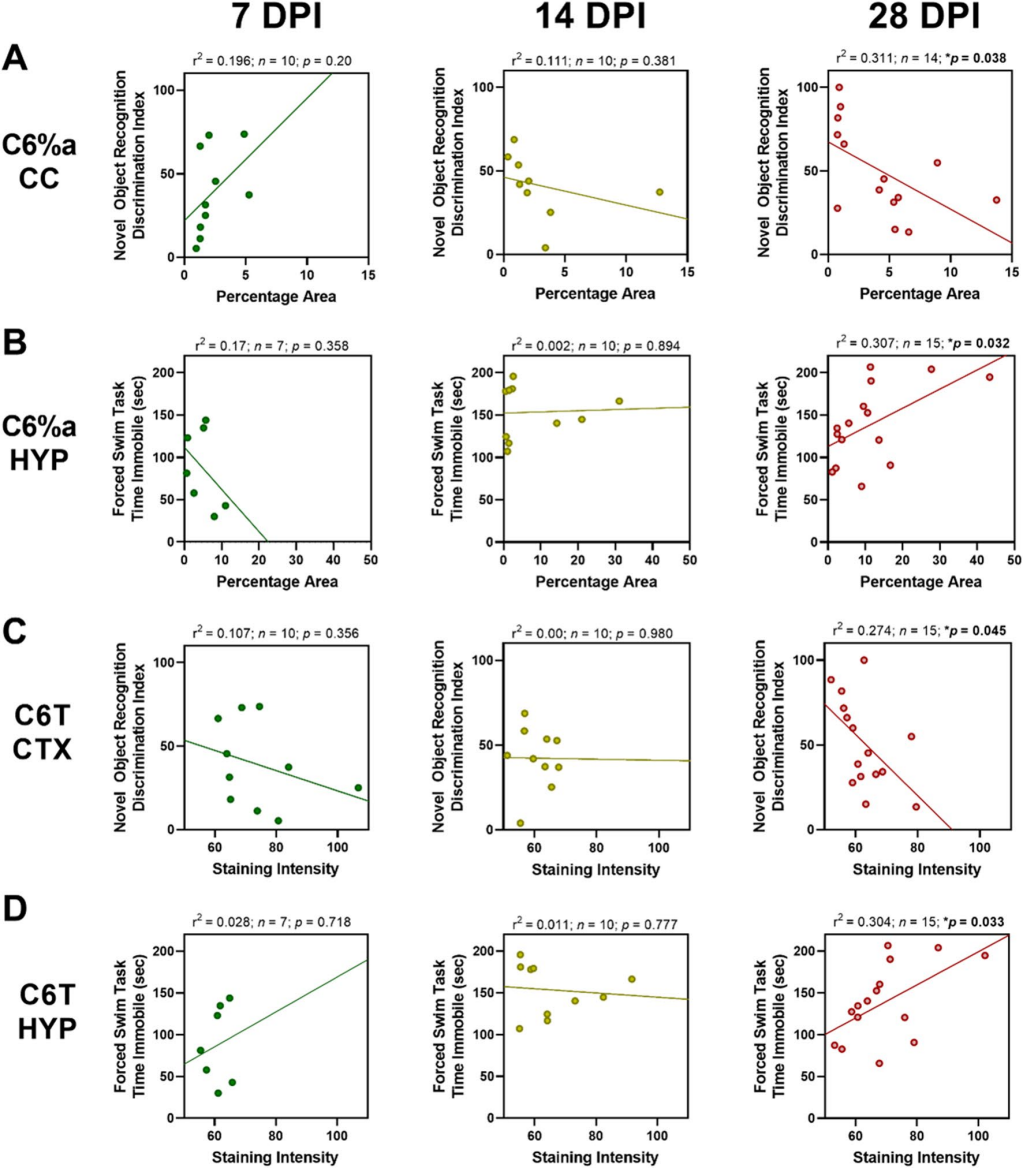
Negative correlations between (**A**) novel object recognition (NOR) task or (**B**) forced swim test (FST) task and C6T Aβ variant staining increased with days post-injury (DPI). (**A**) At 7 and 14 DPI, the discrimination index on the NOR task was not significantly correlated with punctate C6T Aβ variant staining but was significantly correlated with behavioral deficits at 28 DPI in the corpus callosum (CC). (**B**) At 7 and 14 DPI, time spent immobile during the FST was not significantly correlated with punctate C6T Aβ variant staining, but was significantly correlated at 28 DPI in the hypothalamus (HYP). (**C)** At 7 and 14 DPI, the NOR task was not significantly correlated with diffuse C6T Aβ variant staining, but was significantly correlated at 28 DPI in the cortex (CTX). (**D**) At 7 and 14 DPI, FST was not significantly correlated with diffuse C6T Aβ variant staining but was significantly correlated at 28 DPI in the HYP

There were seven significant correlations with behavioral deficits at 28 DPI, all between either the F9T (tau) variant or the C6T (Aβ) variant (Table 2). Levels of the F9T (tau) variant in three different brain regions (CC, HYP, and CST) all correlated with decreased performance in the forced swim task (FST) at 28 DPI. Levels of the C6T (Aβ) variant (both punctate and diffuse) in the HYP correlated with decreased performance in the FST at 28 DPI, and levels of C6T (Aβ) punctate staining in the CC and diffuse staining in the CTX correlated with decreased performance in the novel object recognition (NOR) test at 28 DPI. There were significant correlations between behavioral deficits and the staining intensity of the F9T (tau) variant (FST-Fig. 6) and the C6T (Aβ) variant (FST and NOR-Fig. 7) at 28 DPI. While none of the 7 or 14 DPI correlations between behavior and staining intensity were statistically significant for either the F9T (tau) or C6T (Aβ) variants, all the correlations shifted with time and reached statistical significance by 28 DPI. Specifically, F9T (tau) levels in the CC (Fig. 6A), HYP (Fig. 6B) and CST (Fig. 6C) all correlated negatively with FST at 28 DPI (see Figures for r^2^ and *p*-values). Similarly, for the C6T (Aβ) variant, there were significant correlations with behavioral deficits at 28 DPI. Specifically, punctate C6T (Aβ) staining in the CC (Fig. 7A) correlated negatively with NOR at 28 DPI, and punctate staining in the HYP (Fig. 7B) correlated negatively with FST at 28 DPI (see Figures for r^2^ and *p*-values). Diffuse C6T (Aβ) staining in the CTX (Fig. 7C) correlated negatively with NOR at 28 DPI, and diffuse staining in the HYP (Fig. 7D) correlated negatively with FST at 28 DPI (see Figures for r^2^ and *p*-values).

## Discussion

TBI induces pathophysiological processes in the brain which can generate toxic protein variants associated with neurodegenerative diseases and corresponding behavioral deficits. Neuronal axons are particularly vulnerable to the high sheer stress caused by TBI, which can disrupt protein axonal transport mechanisms including transport of tau [5–9]. Stress from TBI can disrupt cellular proteostasis leading to intracellular generation and accumulation of misprocessed and sometimes toxic protein variants of Aβ, tau, TDP-43, and α-syn. In humans, a TBI is a heterogenous injury that causes a broad range of morbidities including sensorimotor, affective, and cognitive deficits [50]. Clinical data suggest that some injury-induced morbidities may increase with severity of trauma. TBI survivors with similar injury severities experience varied symptoms and impairments. We observed similar individual differences in our mouse cohorts. We controlled for variables such as genetic background, sex, weight, age, and location of the craniectomy. Despite identical injuries, pathological response and recovery were distinctively different in some individuals, resulting in different behavioral outcomes and protein variant profiles between individual mice. Variation in pathological responses of genetically identical mice may be attributed in part to discrete epigenetic differences acquired after conception until sacrifice.

We show that neurodegenerative disease related variants of Aβ, tau, TDP-43, and α-syn are all generated as a pathophysiological response to TBI. Numerous statistically significant differences in protein variant staining levels in regions adjacent to the site of injury compared to control shams were observed at 7 DPI, particularly in the corpus callosum and cortex. However, these differences essentially resolved by 14 and 28 DPI. In contrast, only a single correlation with protein variant accumulation was observed with any of the deeper brain structures; and only at 28 DPI, between the D11C (tau) variant and the hypothalamus. However, we observed that individual mice had elevated levels of selected protein variants in the deeper brain regions, particularly in tissue collected at 28 DPI. These individuals demonstrated a further decline in neurobehavioral testing at more subacute time points. We found 21 statistically significant correlations between protein variants in specific brain regions and behavioral deficits, 13 reflecting acute changes (7 DPI), and seven reflecting subacute changes (28 DPI) in the brain.

The changes in protein variant levels at 7 DPI reflect the disruption of cell proteostasis during the acute phase of recovery from TBI and disruption of the blood brain barrier integrity. Markedly, the A4 (Aβ) variant was disproportionately represented in behavioral deficits associated with the acute phase. Part of the initial acute response to TBI is an increase in neuronal expression of APP and BACE-1, a protease which cleaves APP, leading to increased Aβ levels [51–55]. This acute response to brain injury generates an early transcriptional burst of Aβ by neural cells, resulting in short term Aβ accumulation (see Suppl Fig. 1). This extracellular generation of Aβ has been suggested as a defensive mechanism with several potential functions [56]. First, Aβ can react with fibrin to quickly seal ruptured blood vessels in the blood brain barrier [57] where the microvasculature of the brain is closely associated with amyloid plaques produced after injury [57]. This defensive mechanism is evidenced by the high levels of both Aβ and IgG that accumulate in tissue and vasculature around the site of injury at 7 DPI (See Suppl. Figures 1, 2). Second, Aβ has also been shown to promote synaptogenesis, also a critically important function after brain injury [58]. Notably, the A4 (Aβ) scFv was generated against a small toxic synthetically generated oligomeric Aβ variant [33] that can assemble and accumulate intra- or extracellularly in the brain. The numerous acute phase correlations between A4 (Aβ) levels and behavioral deficits may well reflect Aβ aggregation resulting from the burst of extracellular Aβ generated during the acute response to TBI.

In contrast to the acute post-injury period, during the subacute period, the bulk of the neurodegenerative disease associated protein variants had been cleared from the brain, except for two specific toxic protein variants, the C6T (Aβ) variant [48, 59] and the F9T (tau) variant [31, 60, 61], which accounted for all of the significant correlations between subacute behavioral deficits and protein variant levels. C6T (Aβ) punctate staining in particular showed high staining levels in individual mice in several brain regions distant from the site of injury. Notably, C6T (Aβ) punctate staining levels were also higher in the 2 TBIs compared to 1 TBI groups in several of the deeper brain regions (HYP, AMY and CP), though there is substantial variance between individual mice and the difference did not reach statistical significance. Both the C6T (Aβ) and F9T (tau) variants showed a time dependent progression in their effect on behavioral outcomes where these protein variants positively correlated with acute behavioral tests (7 DPI), with a change toward a negative correlation at 14 DPI, which reached statistically significant negative correlations by 28 DPI.

### Potential mechanistic links between TBI and increased risk of AD

The C6T (Aβ) scFv was generated against an intracellularly generated Aβ variant isolated from human AD brain tissue [59]. The staining patterns of C6T (Aβ) and F9T (tau) in the cytoplasm of pyramidal neurons in TBI mouse brain tissue collected at 28 DPI (Fig. 5C, F) reflects an ongoing disruption of proteostasis. Notably, these same staining patterns are also observed in human post-mortem AD tissue (Fig. 5D, E,G). Cytoplasmic accumulation of C6T reactive Aβ in neurons in the TBI mouse brain tissue at 28 DPI reflects the specificity of the C6T scFv for an intracellularly generated AD related Aβ variant, as these same Aβ variants are also present in the cytoplasm of neurons in early and late stage AD brain tissue (Fig. 5C-E). While the extracellularly generated A4 (Aβ) variant can accumulate as a direct consequence of the initial acute transcriptional response to brain injury, the toxic intracellular C6T (Aβ) variant is produced as a result of neuronal cells still under chronic stress and disrupted proteostasis after resolution of the initial injury response. Similarly, the F9T (tau) variant also correlated with injury and may reflect damage to neuronal axons. The white matter tracts extending from the pyramidal neurons contain high levels of tau and accumulation of neurotoxic F9T (tau) variants in the conductive axons of the corpus callosum can cause disruption of neuronal function [62].

The C6T (Aβ) and F9T (tau) variants correlated with long-term behavioral deficits following TBI and we previously showed that both of these variants are also excellent biomarkers for early detection of AD [37–39]. Therefore, the significant correlation of long-term cognitive deficits with increased levels of C6T (Aβ) and F9T (tau) variants provide compelling evidence for a pathogenic link between unresolved protein pathology resulting from TBI and early stage AD related neurodegeneration. Both the C6T (Aβ) and F9T (tau) variants have been detected in sera samples from human TBI cases; even years after injury [40], lending additional support to the validity of the mouse TBI models for replicating human TBI pathology. Notably none of the α-syn or TDP-43 variants studied correlated significantly with the behavioral tests at 28 DPI. The correlations of the C6T (Aβ) and F9T (tau) variants with long-term cognitive deficits in the TBI mice, along with their presence in sera samples from human TBI cases [40], and sera samples from human post-mortem and longitudinal AD cases [37–39], suggest that there is a common pathogenesis in all of these cases.

These results provide very intriguing insights into the pathologic link between TBI and AD susceptibility and suggests potential biomarkers that can be utilized to identify individual TBI patients at an increased risk for AD. Notably these biomarkers represent generation of earlier stage neuropathology that is topographically quite different from the more advanced plaques and tangles that define AD, and represent promising targets for early disease detection and subsequent therapeutic intervention. We previously demonstrated that selectively targeting the C6T reactive Aβ variant in a mouse model of AD provides excellent therapeutic benefit; restoring neuronal integrity to wild-type levels [48]. Early therapeutic treatment of TBI patients could help reduce harmful acute inflammatory responses and restore neuronal proteostasis. We anticipate that additional correlations between other protein variants and neurobehavioral deficits associated with other neurodegenerative diseases will be identified, and these will be the subjects of further studies.

## Supplementary Information

Below is the link to the electronic supplementary material.

Supplementary file1 (DOCX 27 KB)

Supplementary file2 (PDF 7.07 MB)

## Data Availability

The behavior data supporting the conclusions of this articles are publicly available in the Open Data Commons for Traumatic Brain Injury (ODC-TBI) at the following link: DOI:https://doi.org/10.34945/F5BS3R.

## Abbreviation

AD: Alzheimer’s disease
AD-TDP3: Antibody fragment against TDP-43 variant
AMY: Amygdala/olfactory/cortical subplate region
ARRIVE: Animal Research: Reporting In Vivo Experiments
Aβ: Beta-amyloid
A4: Antibody fragment against oligomeric beta-amyloid
CC: Corpus callosum
CP: Caudoputamen
CST: Corticospinal tract
CTX: Upper cortex
C6T: Antibody fragment against oligomeric beta-amyloid
DI: Discrimination index
DPI: Days post injury
D5: Antibody fragment against oligomeric a-synuclein
D11C: Antibody fragment against oligomeric tau
EPM: Elevated plus maze
FPI: Fluid percussion injury
FST: Forced swim task
FX: Fornix
F9T: Antibody fragment against oligomeric tau
HC: Hippocampus
HYP: Hypothalamus
IgG: Immunoglobulin G
mFPI: Midline fluid percussion injury
NOR: Novel object recognition
NSS: Neurological severity score
RR: Rotarod
scFv: Single chain antibody variable domain
STR: Striatum
TBI: Traumatic brain injury
TDP-43: Transactive response DNA binding protein 43
TH: Thalamus
α-syn: Alpha-synuclein
10H: Antibody fragment against oligomeric a-synuclein
