# Supplementary material for: Traumatic Brain Injury in Mice Generates Early-Stage Alzheimer’s Disease Related Protein Pathology that Correlates with Neurobehavioral Deficits
Source: Mol Neurobiol. 2024 Feb 27;61(10):7567–82. doi: 10.1007/s12035-024-04035-5 (PMC11415463; doi:10.1007/s12035-024-04035-5)
Supplement: Supplementary file 1 — Supplementary file1 (DOCX 27 KB) [file 12035_2024_4035_MOESM1_ESM.docx]

**Supplementary Table 1:** Staining intensity of A4 anti Aβ-variant in multiple brain regions. * Indicates *p* < 0.05.

| **Region** | **F (DF_n_, DF_d_)** | ***P* – value** | ***N*** |
| --- | --- | --- | --- |
| Cortex | F _(6,31)_ = 6.000 | **0.0003** ***** | 38 |
| Corpus Callosum | F _(6,31)_ = 3.387 | **0.011 *** | 38 |
| Hippocampus | F _(6,28)_ = 2.989 | **0.022 *** | 35 |
| Fimbria/Fiber Tract | F _(6,31)_ = 2.278 | 0.062 | 38 |
| Caudoputamen | F _(6,31)_ = 1.645 | 0.168 | 38 |
| Thalamus | F _(6,31)_ = 2.252 | 0.064 | 38 |
| Hypothalamus | F _(6,31)_ = 1.611 | 0.177 | 38 |
| Amygdala/Olfactory | F _(6,31)_ = 1.596 | 0.182 | 38 |

**Supplementary Table 2:** Staining intensity of C6T anti Aβ-variant in multiple brain regions. * Indicates *p* < 0.05.

| **Region** | **F (DF_n_, DF_d_)** | ***P* – value** | ***N*** |
| --- | --- | --- | --- |
| Cortex | F _(6,32)_ = 3.595 | **0.008** ***** | 39 |
| Corpus Callosum | F _(6,30)_ = 1.221 | 0.323 | 37 |
| Striatum | F _(6,28)_ = 1.813 | 0.133 | 35 |
| Fimbria/Fiber Tract | F _(6,26)_ = 2.007 | 0.101 | 33 |
| Caudoputamen | F _(6,31)_ = 0.504 | 0.800 | 38 |
| Thalamus | F _(6,30)_ = 3.220 | **0.015 *** | 37 |
| Hypothalamus | F _(6,32)_ = 1.254 | 0.306 | 39 |
| Amygdala/Olfactory | F _(6,27)_ = 1.174 | 0.349 | 34 |

**Supplementary Table 3:** Percentage area coverage of C6T anti Aβ-variant in multiple brain regions. * Indicates *p* < 0.05.

| **Region** | **F (DF_n_, DF_d_)** | ***P* – value** | ***N*** |
| --- | --- | --- | --- |
| Cortex | F _(6,32)_ = 0.590 | 0.736 | 39 |
| Corpus Callosum | F _(6,30)_ = 0.772 | 0.598 | 37 |
| Striatum | F _(6,28)_ = 0.610 | 0.721 | 35 |
| Fimbria/Fiber Tract | F _(6,26)_ = 1.907 | 0.118 | 33 |
| Caudoputamen | F _(6,30)_ = 0.806 | 0.573 | 37 |
| Thalamus | F _(6,31)_ = 1.100 | 0.385 | 38 |
| Hypothalamus | F _(6,32)_ = 1.023 | 0.428 | 39 |
| Amygdala/Olfactory | F _(6,27)_ = 0.853 | 0.541 | 34 |

**Supplementary Table 4:** Staining intensity of 10H anti-a-synuclein variant in multiple brain regions. * Indicates *p* < 0.05.

| **Region** | **F (DF_n_, DF_d_)** | ***P* – value** | ***N*** |
| --- | --- | --- | --- |
| Cortex | F _(6,32)_ = 8.392 | **< 0.0001 *** | 39 |
| Corpus Callosum | F _(6,32)_ = 3.078 | **0.017 *** | 39 |
| Hippocampus | F _(6,30)_ = 3.500 | **0.010 *** | 37 |
| Corticospinal Tract | F _(6,32)_ = 2.717 | **0.030*** | 39 |
| Caudoputamen | F _(6,32)_ = 1.823 | 0.126 | 39 |
| Thalamus | F _(6,31)_ = 5.374 | **0.0007 *** | 38 |
| Hypothalamus | F _(6,31)_ = 1.127 | 0.370 | 38 |
| Amygdala/Olfactory | F _(6,31)_ = 2.408 | **0.050 *** | 38 |

**Supplementary Table 5:** Staining intensity of D5 anti-a-synuclein variant in multiple brain regions. * Indicates *p* < 0.05.

| **Region** | **F (DF_n_, DF_d_)** | ***P* – value** | ***N*** |
| --- | --- | --- | --- |
| Cortex | F _(6,32)_ = 5.023 | **0.001 *** | 39 |
| Corpus Callosum | F _(6,27)_ = 0.293 | 0.935 | 34 |
| Striatum | F _(6,29)_ = 1.404 | 0.247 | 36 |
| Corticospinal Tract | F _(6,26)_ = 0.412 | 0.864 | 33 |
| Caudoputamen | F _(6,29)_ = 0.349 | 0.905 | 36 |
| Fimbria/Fiber Tracts | F _(6,32)_ = 2.056 | 0.087 | 39 |
| Amygdala/Olfactory | F _(6,32)_ = 3.418 | **0.010 *** | 39 |

**Supplementary Table 6:** Staining intensity of F9T anti-tau variant in multiple brain regions. * Indicates *p* < 0.05.

| **Region** | **F (DF_n_, DF_d_)** | ***P* – value** | ***N*** |
| --- | --- | --- | --- |
| Cortex | F _(6,32)_ = 2.752 | **0.029 *** | 39 |
| Corpus Callosum | F _(6,31)_ = 2.216 | 0.068 | 38 |
| Fimbria/Fiber Tracts | F _(6,26)_ = 0.528 | 0.782 | 33 |
| Corticospinal Tract | F _(6,28)_ = 0.986 | 0.454 | 35 |
| Caudoputamen | F _(6,32)_ = 1.990 | 0.097 | 39 |
| Thalamus | F _(6,31)_ = 0.975 | 0.459 | 38 |
| Hypothalamus | F _(6,31)_ = 1.095 | 0.387 | 38 |
| Amygdala/Olfactory | F _(6,30)_ = 0.866 | 0.531 | 37 |

**Supplementary Table 7:** Staining intensity of D11C anti-tau variant in multiple brain regions. * Indicates *p* < 0.05.

| **Region** | **F (DF_n_, DF_d_)** | ***P* – value** | ***N*** |
| --- | --- | --- | --- |
| Cortex | F _(6,32)_ = 5.574 | **0.0005 *** | 39 |
| Corpus Callosum | F _(6,31)_ = 3.552 | **0.009 *** | 38 |
| Hippocampus | F _(6,29)_ = 4.187 | **0.004 *** | 36 |
| Corticospinal Tract | F _(6,25)_ = 1.376 | 0.263 | 32 |
| Caudoputamen | F _(6,17)_ = 1.024 | 0.443 | 24 |
| Thalamus | F _(6,32)_ = 1.900 | 0.112 | 39 |
| Hypothalamus | F _(6,31)_ = 2.806 | **0.027 *** | 38 |
| Amygdala/Olfactory | F _(6,29)_ = 2.132 | 0.080 | 36 |

**Supplementary Table 8:** Staining intensity of ADTDP3 anti-TDP-43 variant in multiple brain regions. * Indicates *p* < 0.05.

| **Region** | **F (DF_n_, DF_d_)** | ***P* – value** | ***N*** |
| --- | --- | --- | --- |
| Cortex | F _(6,28)_ = 4.178 | **0.040 *** | 35 |
| Corpus Callosum | F _(6,24)_ = 3.014 | **0.024 *** | 31 |
| Hippocampus | F _(6,28)_ = 1.733 | 0.150 | 35 |
| Thalamus | F _(6,__29)_ = 1.845 | 0.125 | 36 |
| Hypothalamus | F _(6,29)_ = 1.404 | 0.247 | 36 |
| Amygdala/Olfactory | F _(6,29)_ = 1.831 | 0.128 | 36 |
