# Supplementary material for: Traumatic Brain Injury in Mice Generates Early-Stage Alzheimer’s Disease Related Protein Pathology that Correlates with Neurobehavioral Deficits
Source: Mol Neurobiol. 2024 Feb 27;61(10):7567–82. doi: 10.1007/s12035-024-04035-5 (PMC11415463; doi:10.1007/s12035-024-04035-5)
Supplement: Supplementary file 2 — Supplementary file2 (PDF 7.07 MB) [file 12035_2024_4035_MOESM2_ESM.pdf]

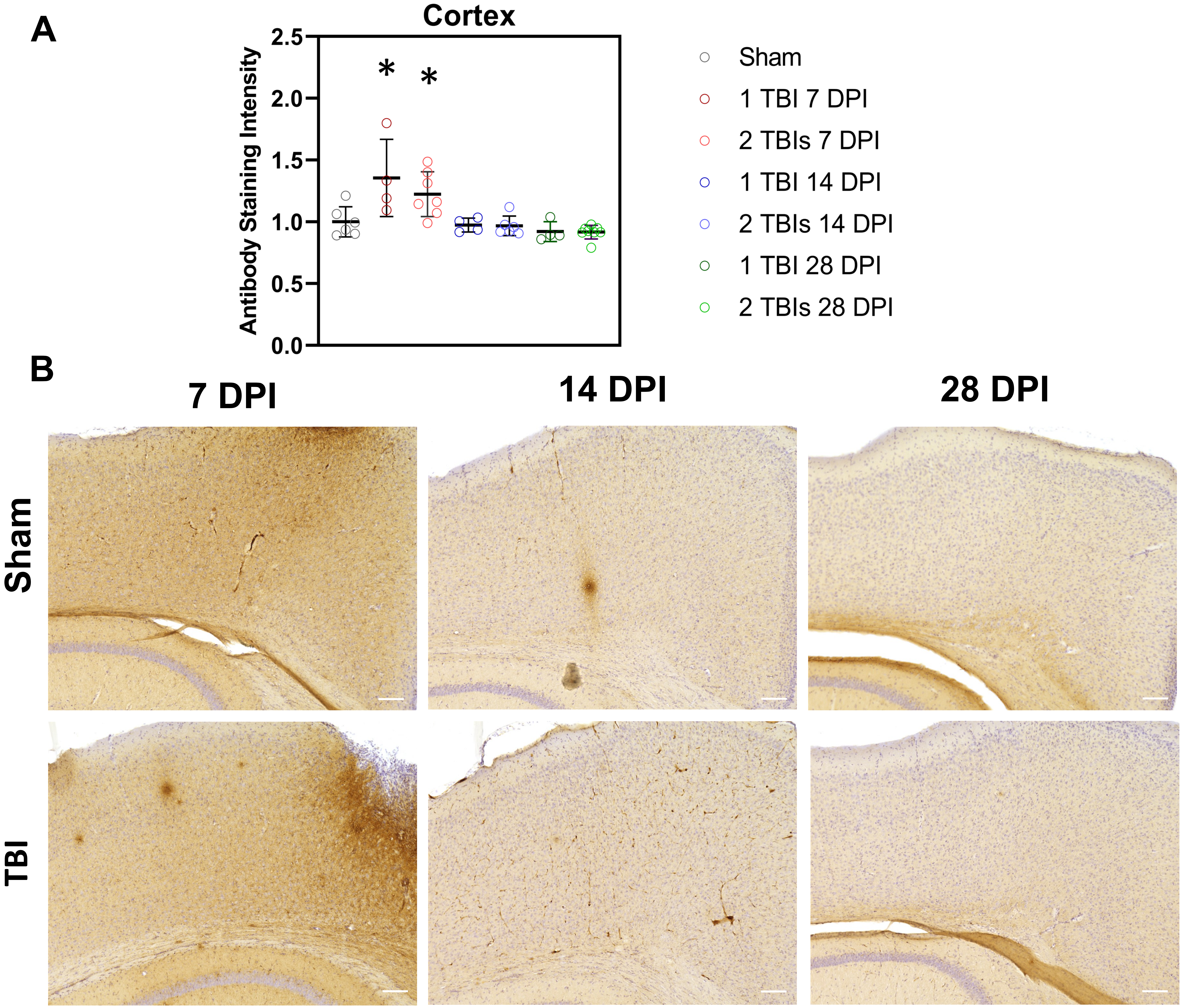

Supplemental Figure 1: (A) Dunnett post hoc analysis of intensity values of amyloid-beta (Aβ) staining of coronal slices of mouse cortex using a commercial anti-Aβ antibody (6E10). Brain tissue was collected at 7, 14, or 28 days post injury (DPI) from mice subjected to a control sham injury, 1 TBI, or 2 TBIs. There were significant differences in the 1 TBI ($p = 0.0027$) and 2 TBI ($p = 0.0377$) groups at 7 DPI compared to shams. Asterisks indicate $p < 0.05$ significance compared to corresponding sham region. Error bars are mean ± SD. **(B)** Representative coronal sections stained with commercial Aβ antibody (6E10) grouped by sham (craniectomy without TBI; top) or TBI (bottom). Primary antibody was detected with HRP conjugated secondary antibody/DAB (brown) and nuclei detected with hematoxylin (purple). The most intense staining was observed near the site of craniectomy and TBI in tissue collected at 7 DPI, resolving by 14 and 28 DPI. Scale bar 100 µm.

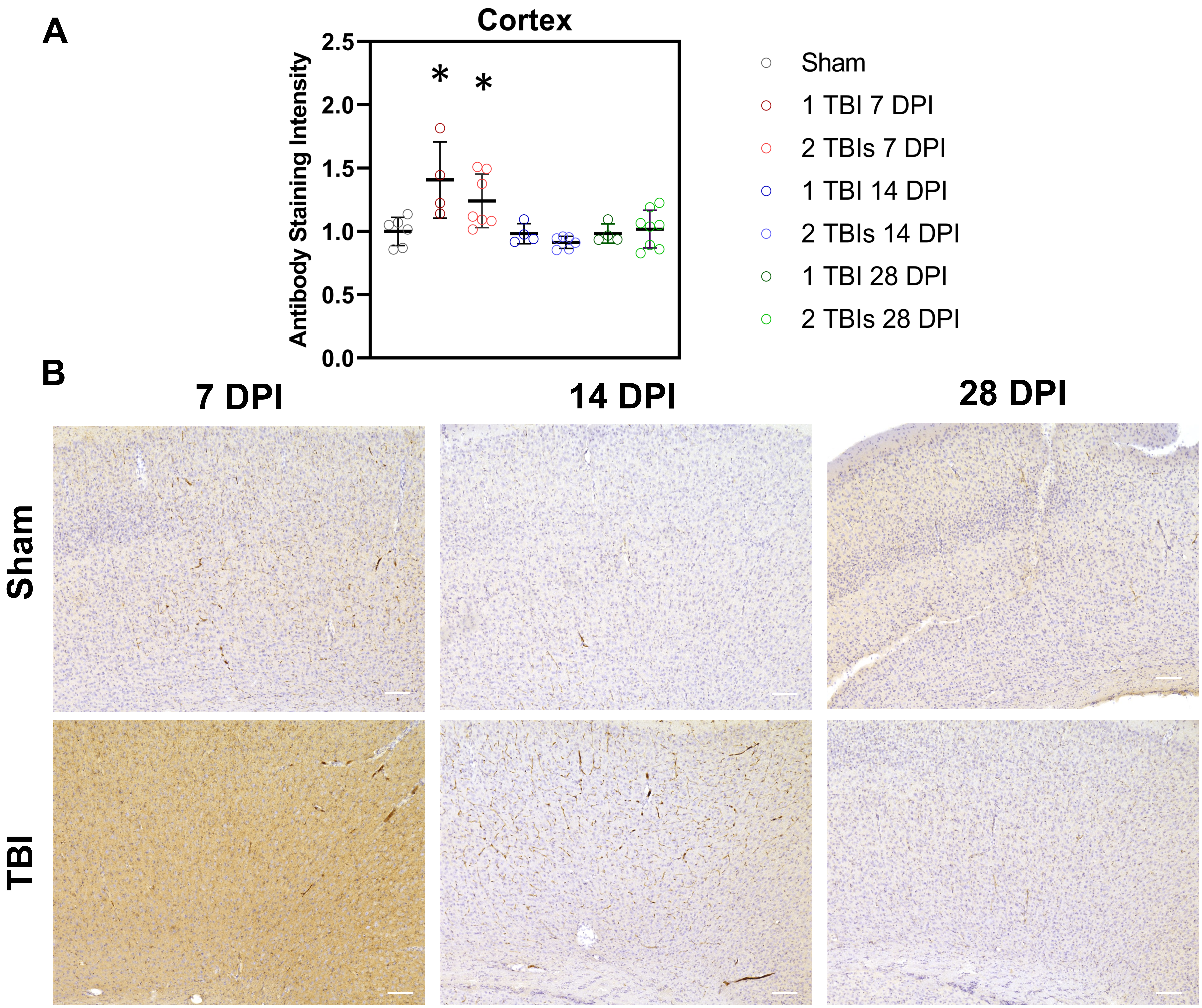

Supplemental Figure 2: (A) Dunnett post hoc analysis of immunoglobulin G (IgG) staining intensity. Brain tissue was collected at 7, 14, or 28 days post injury (DPI) from mice subjected to a control sham injury, 1 TBI, or 2 TBIs. There were significant differences in the 1 TBI (p = 0.0021) and 2 TBI (p = 0.0486) groups at 7 DPI compared to sham group. Asterisks indicate $p < 0.05$ significance compared to corresponding sham region. Error bars are mean ± SD. **(B)** Representative coronal sections stained with an anti-mouse IgG primary antibody grouped by control sham (craniectomy without TBI; top) or TBI (bottom). Antibody was detected with HRP conjugated secondary antibody/DAB (brown) and nuclei detected with hematoxylin (purple). Intense staining was observed near the site of craniectomy and TBI in tissue collected at 7 DPI, resolving by 14 and 28 DPI. Scale bar 100 µm.

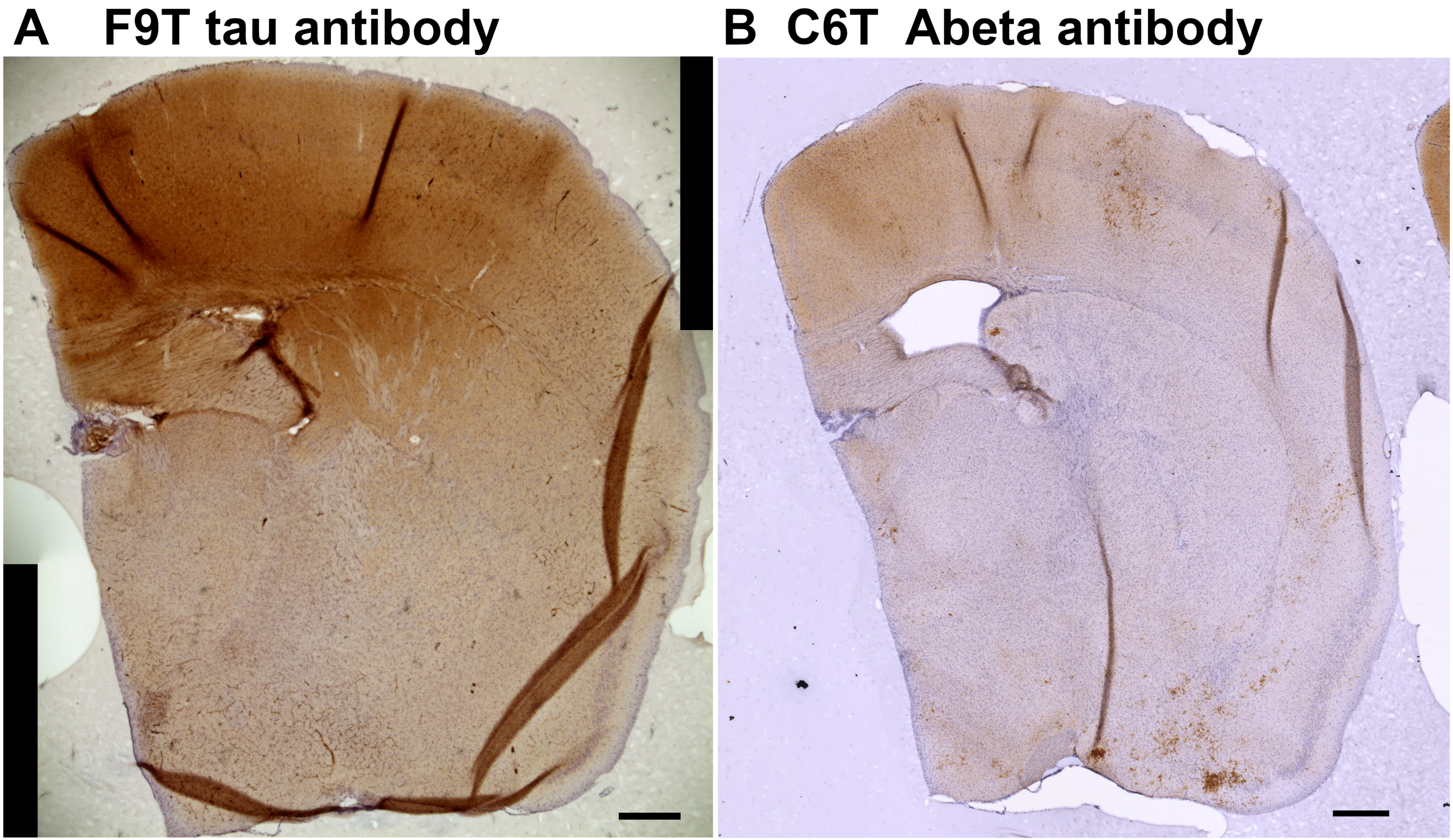

Supplemental Figure 3: Representative coronal mouse brain tissue staining detected with (A) F9T anti tau scFv-phage and (B) C6T anti-Aβ scFv-phage. Tissue was collected at 7 days post-injury (DPI). Phage were detected with HRP conjugated secondary/DAB (brown) and nuclei detected with hematoxylin (purple). While staining was observed throughout the brain, the most intense staining was observed near the site of craniectomy. Scale bar 500 μm.

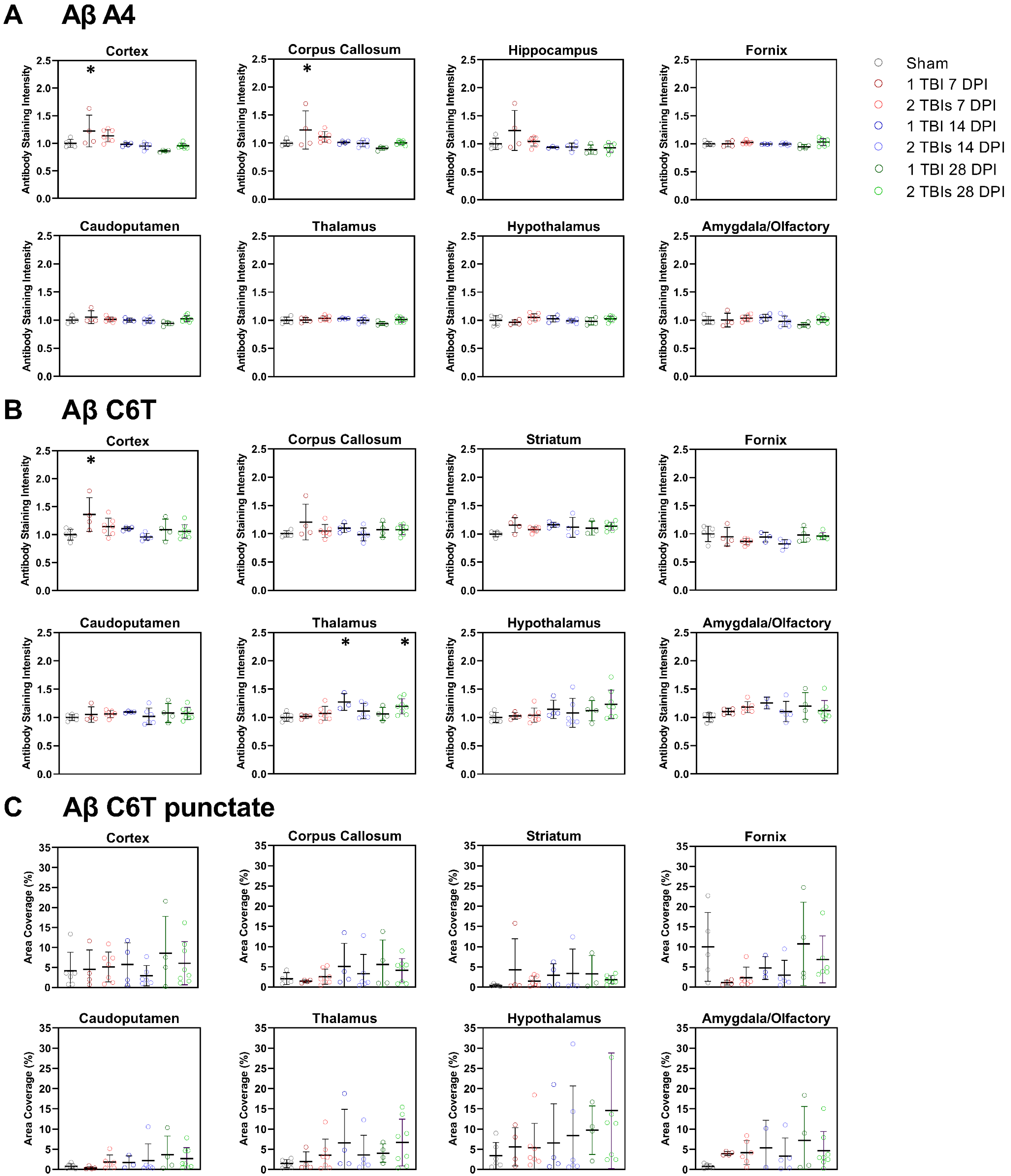

Supplemental Figure 4: Dunnett post hoc analysis of intensity values of amyloid-beta (Aβ) variant staining using ScFv-phage targeting two different Aβ variants: **(A)** oligomeric A4 Aβ variant; and **(B)** diffuse and **(C)** punctate staining patterns by human AD brain derived C6T Aβ variant. Diffuse staining intensity and percent area covered by punctate staining were separately analyzed for each brain region using ImageJ. Brain tissue was collected at 7, 14, or 28 DPI from mice subjected to a control sham injury, 1 TBI, or 2 TBIs. Staining with the A4 phage in the cortex displayed significant differences in the 1 TBI group at 7 DPI (p = 0.023) compared to shams. There were significant differences in the corpus callosum region in the 1 TBI group at 7 DPI (p = 0.0313) compared to corresponding sham group. The C6T phage had significantly higher staining intensities in the cortex between the 1 TBI group at 7 DPI (p = 0.0034) compared to sham group. The thalamus showed significant staining in the 1 TBI group at 14 DPI (p = 0.0125) and 2 TBI group at 28 DPI (p = 0.0215) compared to sham groups. The percentage area of C6T punctate staining did not show significant differences to sham in any of the brain regions of interest. Asterisks indicate $p < 0.05$ compared to corresponding sham regions. Error bars mean ± SD.

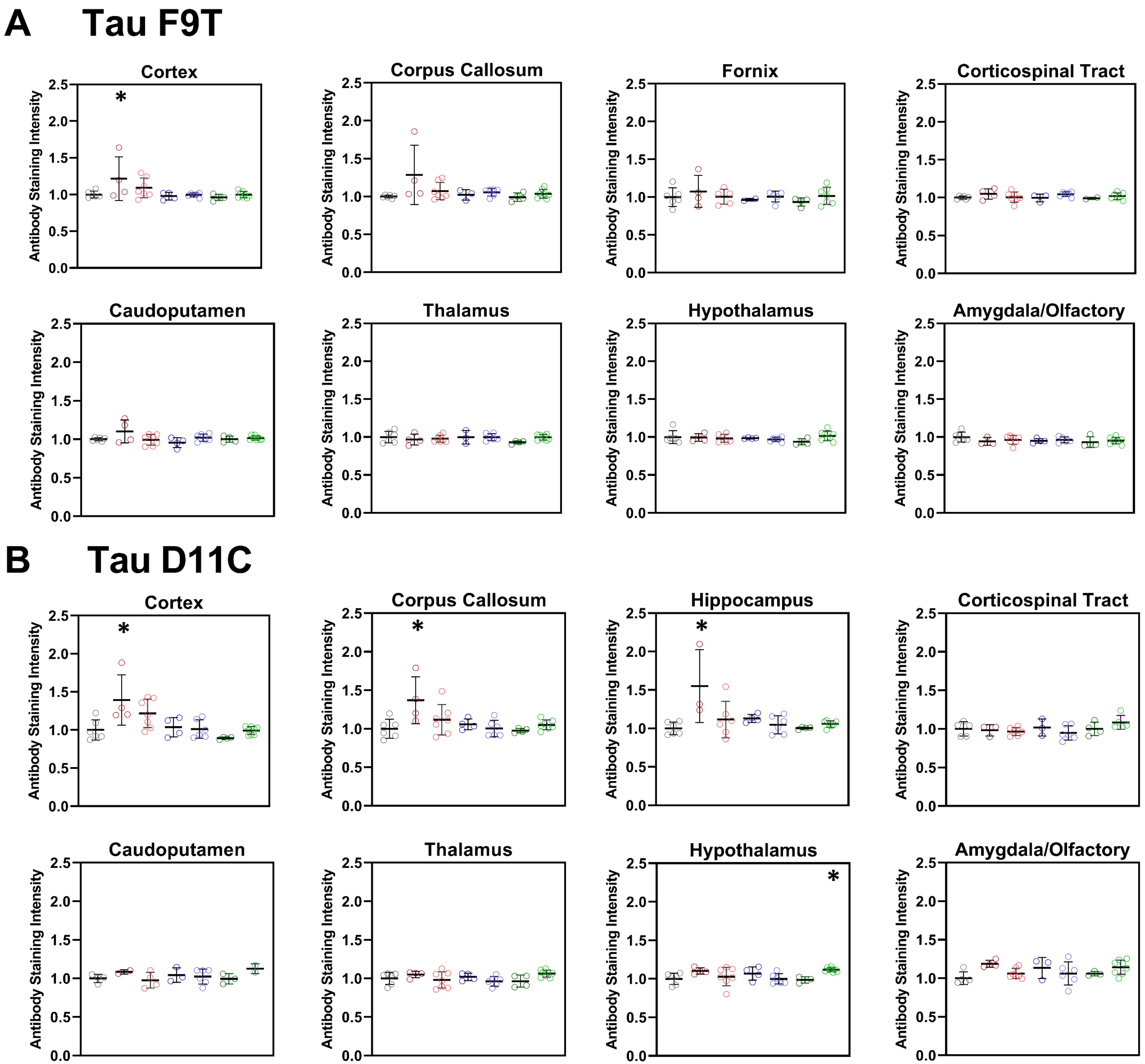

Supplemental Figure 5: Dunnett post hoc analysis of intensity values of tau variant staining of coronal slices of mouse brains using ScFv-phage targeting two different oligomeric tau variants: **(A)** F9T and **(B)** D11C. Brain tissue was collected at 7, 14, or 28 DPI from mice subjected to a control sham injury, 1 TBI, or 2 TBIs. Staining with the F9T phage showed significant differences in the cortex region between the 1 TBI group at 7 DPI ($p = 0.031$) compared to shams. Staining with the D11C phage showed significant differences between the 1 TBI group at 7 DPI in the cortex ($p = 0.002$), corpus callosum, ($p = 0.0029$), and hippocampus ($p = 0.0005$) compared to shams, as well as the hypothalamus in the 2 TBI group at 28 DPI ($p = 0.0325$) compared to sham. Asterisks indicate $p < 0.05$ significance compared to corresponding sham region.

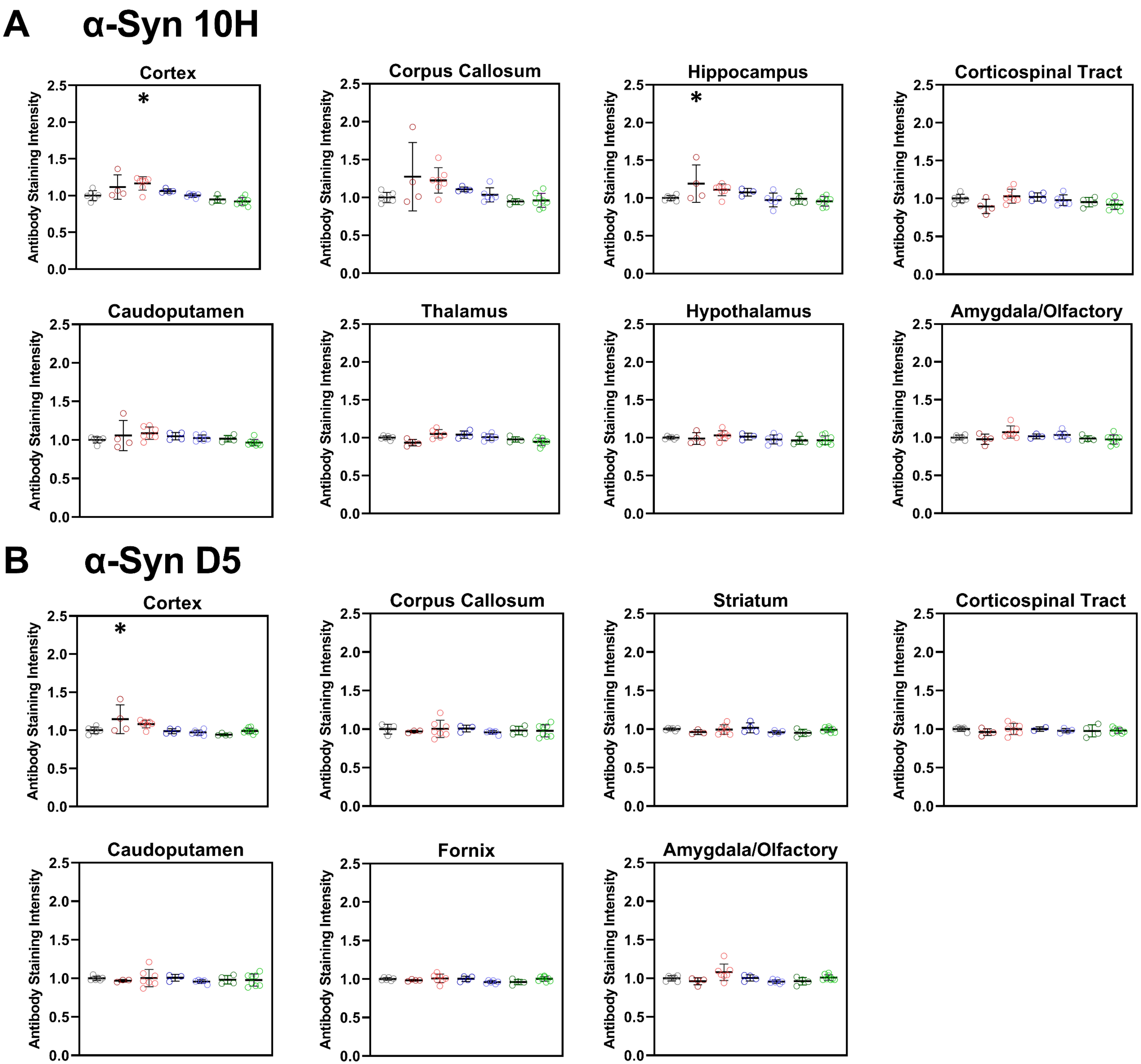

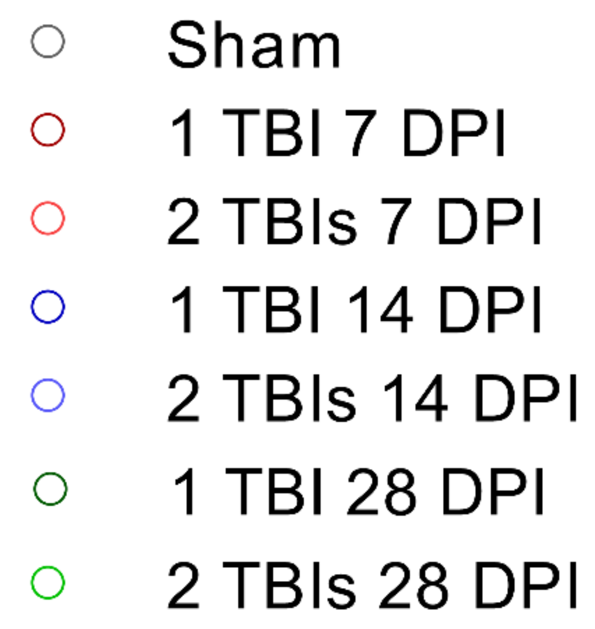

Supplemental Figure 6: Dunnett post hoc analysis of intensity values of alpha-synuclein (a-syn) variant staining using ScFv-phage targeting two different a-syn variants: **(A)** 10H oligomeric a-syn variant; and **(B)** D5 oligomeric a-syn variant. Brain tissue was collected at 7, 14, or 28 DPI from mice subjected to a control sham injury, 1 TBI, or 2 TBIs. Staining with the 10H phage showed significant differences in the cortex region between the 2 TBI group at 7 DPI (p = 0.003) compared to shams. 10H phage staining also showed significant differences in the hippocampus between the 1 TBI group at 7 DPI (p = 0.035) compared to shams. Staining with D5 phage showed significant differences in the CTX between the 1 TBI group at 7 DPI compared to shams (p = 0.03). Asterisks indicate $p < 0.05$ significance to corresponding sham region. Error bars are mean ± SD.

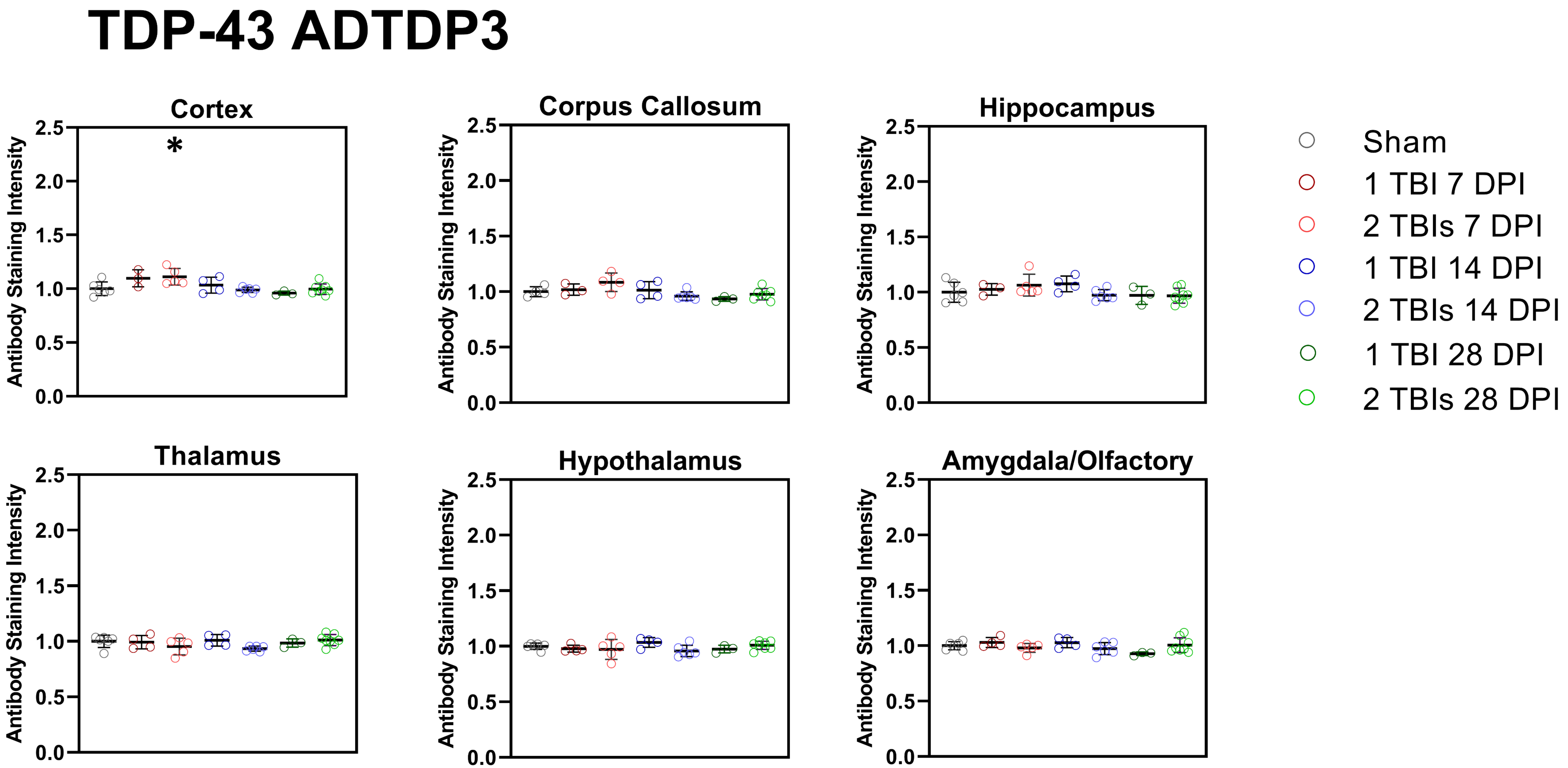

Supplemental Figure 7: Dunnett post hoc analysis of intensity values of TDP-43-variant staining of using AD-TDP3-phage targeting a human ALS brain derived TDP-43 variant. Brain tissue was collected at 7, 14, or 28 DPI from mice subjected to a control sham injury, 1 TBI, or 2 TBIs. Staining with the AD-TDP3 phage showed significant differences in the cortex between the 2 TBI group at 7 DPI and shams ($p = 0.0185$). Asterisks indicate $p < 0.05$ significance compared to the corresponding sham region.
